# Supplementary material for: Methods for estimating the burden of acute tropical infectious diseases: A scoping review
Source: PLoS Negl Trop Dis. 2026 May 4;20(5):e0013359. doi: 10.1371/journal.pntd.0013359 (PMC13160447; doi:10.1371/journal.pntd.0013359)
Supplement: S2 Table — (DOCX) [file pntd.0013359.s002.docx]

**S2 Table. Full search strategy.**

| **Database** | **Search string** |
| --- | --- |
| PubMed | ("Global Burden of Disease"[Mesh] OR "Disability-Adjusted Life Years"[Mesh] OR "Disease burden"[Text Word] OR "burden of disease"[Text Word] OR "disability-adjusted life years"[Text Word] OR "health burden"[Text Word] OR GBD[Text Word] OR "burden estimate*"[Text Word] OR "estimated burden"[Text Word]) AND (("Vector Borne Diseases"[Mesh]) OR ("Zoonoses"[Mesh])) |
