# Supplementary material for: Methods for estimating the burden of acute tropical infectious diseases: A scoping review
Source: PLoS Negl Trop Dis. 2026 May 4;20(5):e0013359. doi: 10.1371/journal.pntd.0013359 (PMC13160447; doi:10.1371/journal.pntd.0013359)
Supplement: S3 Table — (DOCX) [file pntd.0013359.s003.docx]

**S3 Table. Comprehensive list of studies included in the review, with ID corresponding to citation number in the main reference list (n = 60).**

| **ID** | **Authors** | **Title** | **Year** | **Location conducted** | **WHO regions** | **Disease studied** | **Spatial resolution** | **Spatial extent** | **Burden measure** |
| --- | --- | --- | --- | --- | --- | --- | --- | --- | --- |
| 32 | Greenwood, B M et al. | Mortality and morbidity from malaria among children in a rural area of The Gambia, West Africa | 1987 | Gambia | African Region | malaria | village | a rural place | morbidity, mortality |
| 33 | Snow, R W et al. | Models to predict the intensity of Plasmodium falciparum transmission: applications to the burden of disease in Kenya. | 1998 | Kenya | African Region | malaria | administrative level 4 | country | case number, death number |
| 34 | Snow, R W et al. | Estimating mortality, morbidity and disability due to malaria among Africa's non-pregnant population. | 1999 | Africa | African Region | malaria | study-site-level | multiple countries | morbidity, mortality, disability |
| 83 | Crump, John A et al | Estimating the incidence of typhoid fever and other febrile illnesses in developing countries | 2003 | lower Egypt | Eastern Mediterranean Region | typhoid fever and brucellosis | district | single district | incidence |
| 35 | Roca-Feltrer, Arantxa et al. | Estimates of the burden of malaria morbidity In Africa in children under the age of five years | 2005 | Africa | African Region | malaria | study-site-level | multiple countries | incidence |
| 36 | Hay, Simon I et al. | Urbanization, malaria transmission and disease burden in Africa. | 2005 | Africa | African Region | malaria | study-site-level | multiple countries | death number |
| 37 | Snow, Robert W et al. | The global distribution of clinical episodes of Plasmodium falciparum malaria. | 2005 | Global | Global | malaria | study-site-level | global | case number |
| 38 | Rowe, Alexander K et al. | The burden of malaria mortality among African children in the year 2000 | 2006 | Africa | African Region | malaria | study-site-level | multiple countries | death number |
| 47 | Anderson, Katie B et al. | Burden of symptomatic dengue infection in children at primary school in Thailand: a prospective study | 2007 | a primary school in Kamphaeng Phet province in northern Thailand | South-East Asian Region; Western Pacific Region | dengue | school | a school | DALYs |
| 48 | Luz, P M et al. | Disability adjusted life years lost to dengue in Brazil | 2009 | Brazil | American | dengue | city and country | country | DALY |
| 84 | Beauté, Julien, and Sirenda Vong. | Cost and disease burden of Dengue in Cambodia | 2010 | Cambodia | Western Pacific region | dengue | community | country | case, deaths, DALYs, economic costs |
| 39 | Hay, Simon I et al. | Estimating the global clinical burden of Plasmodium falciparum malaria in 2007. | 2010 | Global | Global | malaria | study-site-level | global | case number |
| 49 | Wichmann, Ole et al. | Dengue in Thailand and Cambodia: an assessment of the degree of underrecognized disease burden based on reported cases. | 2011 | Thailand and Cambodia | South-East Asian Region; Western Pacific Region | dengue | study-site-level | multiple countries | case number, hospitalized children number |
| 12 | Cibulskis, Richard E et al. | Worldwide Incidence of Malaria in 2009: Estimates, Time Trends, and a Critique of Methods | 2011 | Global | Global | malaria | study-site-level | global | case number, incidence |
| 75 | Alvar, Jorge et al. | Leishmaniasis worldwide and global estimates of Its Incidence | 2012 | Global | Global | leishmaniasis | survey-site-base | global | case number, death number |
| 40 | Gosoniu, Laura et al. | Spatially explicit burden estimates of malaria in Tanzania: bayesian geostatistical modeling of the malaria indicator survey data. | 2012 | Tanzania | African Region | malaria | survey-site-base | country | prevalence, case number |
| 50 | Shepard, Donald S et al. | Economic and disease burden of dengue in Southeast Asia. | 2013 | 12 countries in SEA | African Region | dengue | survey-site-base | multiple countries | case number, death number, DALYs |
| 17 | Undurraga, Eduardo A et al. | Use of expansion factors to estimate the burden of dengue in Southeast Asia: a systematic analysis. | 2013 | 12 countries in SEA | African Region | dengue | study-site-level | multiple countries | case number |
| 79 | Bhatt, Samir et al. | The global distribution and burden of dengue. | 2013 | Global | Global | dengue | point-level | global | infection number |
| 89 | Biggs, Holly M et al. | Estimating leptospirosis incidence using hospital-based surveillance and a population-based health care utilization survey in Tanzania | 2013 | Tanzania | African Region | leptospirosis | survey-site-base | country | incidence |
| 41 | Crowell, Valerie et al. | A novel approach for measuring the burden of uncomplicated Plasmodium falciparum malaria: application to data from Zambia. | 2013 | Zambia | African Region | malaria | national level | country | malaria-attributable fever days per child-year |
| 61 | Garske, Tini et al. | Yellow fever in Africa: estimating the burden of disease and impact of mass vaccination from outbreak and serological data. | 2014 | Africa | African Region | yellow fever | survey-site-base | multiple countries | estimated probability of at least one yellow fever report, force of infection, case number, death number |
| 51 | Shepard, Donald S et al. | Economic and Disease Burden of Dengue Illness in India | 2014 | India | South-East Asian Region | dengue | survey-site-base | country | case number |
| 42 | Griffin, Jamie T et al. | Estimates of the changing age-burden of Plasmodium falciparum malaria disease in sub-Saharan Africa | 2014 | Sub-Sharan Africa | African Region | malaria | continent | multiple countries | clinical incidence, case number |
| 74 | Costa, Federico et al. | Global morbidity and mortality of leptospirosis: A systematic review. | 2015 | Global | Global | leptospirosis | study-site-level | global | incidence, mortality |
| 52 | Undurraga, Eduardo A et al. | Economic and disease burden of dengue in Mexico | 2015 | Mexico | American | dengue | survey-site-base | country | infection number, DALY |
| 53 | Edillo, Frances E et al. | Economic cost and burden of dengue in the Philippines | 2015 | Phillippines | Western Pacific Region | dengue | study-site-level | country | case number |
| 76 | Limmathurotsakul, Direk et al. | Predicted global distribution of Burkholderia pseudomallei and burden of melioidosis | 2016 | Global | Global | melioidosis | study-site-level | global | occurrence risk map, incidence, mortality |
| 14 | Gething, Peter W et al. | Mapping plasmodium falciparum mortality in Africa between 1990 and 2015. | 2016 | Sub-Sharan Africa | African Region | malaria | study-site-level | multiple countries | mortality |
| 81 | Maze, Michael J et al. | Comparison of the estimated incidence of acute leptospirosis in the Kilimanjaro region of Tanzania between 2007–08 and 2012–14 | 2016 | Tanzania | African Region | leptospirosis | district | two districts | incidence |
| 43 | Okami, Suguru, and Naohiko Kohtake. | Fine-scale mapping by spatial risk distribution modeling for regional malaria endemicity and its implications under the low-to-moderate transmission setting in western Cambodia. | 2016 | western Cambodia | Western Pacific Region | malaria | health operational district | multiple provinces | standardized morbidity ratio (SMR)/rilative risk |
| 55 | Wahyono, T Y M et al. | Indonesian dengue burden estimates: review of evidence by an expert panel. | 2017 | Indonesia | South-East Asian Region | dengue | country | country | case number |
| 56 | Undurraga, Eduardo A et al. | Disease burden of dengue in the Philippines: adjusting for underreporting by comparing active and passive dengue surveillance in Punta Princesa, Cebu City. | 2017 | Punta Princesa, Cebu City of Philippines | Western Pacific Region | dengue | city | city | case number, DALY |
| 44 | Camponovo, Flavia et al. | Incidence and admission rates for severe malaria and their impact on mortality in Africa. | 2017 | Sub-Sharan Africa | African Region | malaria | country | multiple countries | severe malaria admission rates, severe disease and mortality rates |
| 62 | Shearer, Freya M et al. | Existing and potential infection risk zones of yellow fever worldwide: a modelling analysis | 2018 | 47 countries across the Americas and Africa | African Region; Region of the Americas | yellow fever | study-site-level | multiple countries | case number |
| 57 | Woon, Yuan Liang et al. | Estimating dengue incidence and hospitalization in Malaysia, 2001 to 2013. | 2018 | Malaysia | Western Pacific Region | dengue | country | country | incidence, infection number, hospitalization rates |
| 18 | Salje, Henrik et al. | Nationally-representative serostudy of dengue in Bangladesh allows generalizable disease burden estimates. | 2019 | Bangladesh | South-East Asian Region | dengue | study-site-level | country | case number, incidence |
| 13 | Weiss, Daniel J et al. | Mapping the global prevalence, incidence, and mortality of Plasmodium falciparum, 2000-17: a spatial and temporal modelling study. | 2019 | Global | Global | malaria | study-site-level and admin polygon | global | incidence, mortality |
| 45 | Battle, Katherine E et al. | Mapping the global endemicity and clinical burden of Plasmodium vivax, 2000-17: a spatial and temporal modelling study. | 2019 | Global | Global | malaria | study-site-level and admin polygon | global | incidence, case number |
| 58 | O’Reilly, Kathleen M., et al. | Estimating the burden of dengue and the impact of release of wMel Wolbachia-infected mosquitoes in Indonesia: a modelling study. | 2019 | Indonesia | South-East Asian Region | dengue | study-site-level | country | case number, DALY |
| 72 | Quan, Tran Minh et al. | Estimates of the global burden of Japanese encephalitis and the impact of vaccination from 2000-2015. | 2020 | 30 endemic area | Global | Japanese encephalitis | study base and admin polygon | multiple countries | case number, death number |
| 92 | Gaythorpe, Katy Am et al. | The effect of climate change on yellow fever disease burden in Africa. | 2020 | Africa | African Region | yellow fever | province | multiple countries continental Africa | death |
| 65 | Mora-Salamanca, Andrés Felipe et al. | Estimating the burden of arboviral diseases in Colombia between 2013 and 2016. | 2020 | Colombia | Americas | dengue, chikungunya, and Zika | administrative level 1 | country | incidence, mortality, DALYs |
| 59 | Cattarino, Lorenzo et al. | Mapping global variation in dengue transmission intensity. | 2020 | Global | Global | dengue | study-site-level | global | case number |
| 94 | Perkins, T Alex et al. | Burden is in the eye of the beholder: Sensitivity of yellow fever disease burden estimates to modeling assumptions | 2021 | Africa | African Region | yellow fever | administrative level 1 | multiple countries | death number |
| 64 | Gaythorpe, Katy Am et al. | The global burden of yellow fever. | 2021 | Africa and South America | African Region; Region of the Americas | yellow fever | study-site-level | multiple countries | (severe) infection number, death number |
| 82 | Pisharody, Sruti et al. | Incidence estimates of acute q fever and spotted fever group rickettsioses, Kilimanjaro, Tanzania, from 2007 to 2008 and from 2012 to 2014 | 2021 | Tanzania | African region | Q fever, spotted fever group | hospital base and admin polygon | two districts | incidence |
| 100 | Wang, Tao et al. | Mapping the distributions of mosquitoes and mosquito-borne arboviruses in China. | 2022 | China | Western Pacific Region | dengue, Japanese encephalitis | study-site-level and admin polygon | country | incidence |
| 46 | Lucas, Tim CD, et al. | Improving disaggregation models of malaria incidence by ensembling non-linear models of prevalence. | 2022 | Madagascar, Colombia, Indonesia and Senegal | African Region; Region of the Americas; South-East Asian Region | malaria | study-site–level, administrative shp | multiple countries | incidence |
| 80 | Luo, Can et al. | Mapping schistosomiasis risk in Southeast Asia: a systematic review and geospatial analysis | 2023 | Southeast Asia | South-East Asian Region | schistosomiasis | survey-site-base | multiple countries | case number |
| 70 | Aiemjoy, Kristen et al. | Estimating the seroincidence of scrub typhus using antibody dynamics after infection | 2024 | Chiang Rai, Thailand, and Vellore, India, Kathmandu Valley, Nepal and Tamil Nadu India | South-East Asian Region | scrub typhus | study-site-level | community level | seroincidence |
| 66 | de Roo, Adrianne Marije et al. | The global health and economic burden of chikungunya from 2011 to 2020: a model-driven analysis on the impact of an emerging vector-borne disease | 2024 | Global | Global | chikungunya | country | global | DALYs |
| 95 | Hartner, Anna-Maria et al. | Estimating the health effects of COVID-19-related immunisation disruptions in 112 countries during 2020-30: a modelling study | 2024 | Global | Global | measles, rubella, hepatitis B, human papillomavirus [HPV], meningitis A, and yellow fever | country, administrative level 1 | global | death number |
| 77 | Schmit, Nora et al. | The public health impact and cost-effectiveness of the R21/Matrix-M malaria vaccine: a mathematical modelling study | 2024 | SSA | African Region | malaria | survey-site-base | regional | case number, death number, DALYs |
| 67 | Kang, Hyolim et al. | Global, regional and national burden of chikungunya: force of infection mapping and spatial modelling study | 2025 | Global | Global | chikungunya | survey-site-base | global | incidence, DALYs, infection number, death number |
| 85 | Ouyang, Han-Qi et al. | Global estimation of dengue disability weights based on clinical manifestations data | 2025 | Global | Global | dengue | hospital base | global | YLD |
| 68 | Ribeiro Dos Santos, Gabriel et al. | Global burden of chikungunya virus infections and the potential benefit of vaccination campaigns | 2025 | Global | Global | chikungunya | 5km for mosquito and population data, country for seroprevalen data | global | case number, death number, DALYs |
| 69 | Pérez-Estigarribia, Pastor E et al. | Modeling the impact of vaccine campaigns on the epidemic transmission dynamics of chikungunya virus outbreaks | 2025 | Paraguay | Region of the Americas | chikungunya | subnational | national | case number, morbidity |
| 78 | Shen, Jing et al. | Vaccination strategies, public health impact and cost-effectiveness of dengue vaccine TAK-003: A modeling case study in Thailand | 2025 | Thailand | South-East Asian Region | dengue | national | country | case number, death number, DALYs |
| 71 | Oo, Win Thandar et al. | Estimating scrub typhus and murine typhus incidence among adolescents and adults in Yangon, Myanmar | 2025 | Yangon of Myanmar | South-East Asian Region | scrub typhus, murine typhus and spotted fever group rickettsioses | household base | state | incidence |
