# Supplementary material for: Methods for estimating the burden of acute tropical infectious diseases: A scoping review
Source: PLoS Negl Trop Dis. 2026 May 4;20(5):e0013359. doi: 10.1371/journal.pntd.0013359 (PMC13160447; doi:10.1371/journal.pntd.0013359)
Supplement: S4 Table — (DOCX) [file pntd.0013359.s004.docx]

**S4 Table. Key information extracted from included studies, with ID corresponding to citation number in the main reference list (n = 60).**

| **ID** | **Title** | **Model** | **Base data** | | **Base data source** | | **Covariates** | **Uncertainty evaluation technique** | |  |
| --- | --- | --- | --- | --- | --- | --- | --- | --- | --- | --- |
| 32 | Mortality and morbidity from malaria among children in a rural area of The Gambia, West Africa | Mortality estimation, morbidity surveillance, microscopy diagnostics, attributable fraction calculations | Mortality | | interview and official records | | seasonal variation in rainfall, age-specific incidence and mortality, availability and utilization of healthcare services, presence of malaria parasites in bloods | Limited; 95% confidence intervals for population attributable fractions using Walter’s method; chi-square tests, Mantel–Haenszel tests, and ANOVA for statistical comparisons; no formal uncertainty propagation for mortality or incidence estimates. | |  |
| 33 | Models to predict the intensity of Plasmodium falciparum transmission: applications to the burden of disease in Kenya. | fuzzy logic climate suitability model | malaria parasite surveys data | | surveillance | | climate variables (rainfall, temperature), NDVI (Normalized Differential Vegetation Index) | Cross-validation using a jack-knife approach, estimation of error rates for endemicity classifications | |  |
| 34 | Estimating mortality, morbidity and disability due to malaria among Africa's non-pregnant population. | fuzzy logic climate suitability model for climate suitability for stable transmission, age-risk function curve using a reciprocal quadratic function to model risks through childhood into adulthood | occurrence, case reporting of malaria, and civil registration data | | literature, active surveillance official report, expert | | temperature and rainfall, population density, age structure, correction factor | Median and interquartile ranges derived from empirical studies; deterministic propagation of IQRs to generate ranges of burden estimates; scenario-based sensitivity via alternative climate, access, and epidemiological assumptions; no probabilistic simulation or Bayesian uncertainty modelling. | |  |
| 83 | Estimating the incidence of typhoid fever and other febrile illnesses in developing countries | incidence estimation system using multipliers: provider-sampling adjustment, diagnostic test sensitivity, seasonality | household survey (health-seeking behaviour, fever prevalence), sentinel surveillance, diagnostic testing data | | health office, survey, lab, surveillance providers | | incidence correction: health-seeking behaviour proportions, provider type, diagnostic test sensitivity, seasonality, age | Deterministic adjustment using fixed multipliers for provider sampling, diagnostic sensitivity, and seasonality; uncertainty addressed through stated assumptions and qualitative discussion; no confidence intervals or probabilistic uncertainty analysis. | |  |
| 35 | Estimates of the burden of malaria morbidity In Africa in children under the age of five years | random effects Poisson regression analysis | malaria morbidity outcome, characteristics of each study and population | | literature, surveillance | | study-level and population characteristics | Jackknife resampling to estimate standard errors; Monte Carlo simulation (10,000 runs) to generate 95% uncertainty intervals for country-level incidence estimates; random-effects Poisson regression for non-severe malaria; interquartile ranges (IQRs) used for severe outcomes where data were sparse. | |  |
| 36 | Urbanization, malaria transmission and disease burden in Africa. | combination of spatial epidemiology models, which included APfEIR (annual P. falciparum entomological inoculation rates) and PR (parasite prevalence ratio) data | PR survey data and APfEIR observations, malaria  mortality | | national demographic, surveys, literature | | climate suitability, urban extent maps and census data | - | |  |
| 37 | The global distribution of clinical episodes of Plasmodium falciparum malaria. | incidence times population at risk based on different risk limits | malaria morbidity, disability and mortality in populations living under different transmission intensity risks | | literature | | clipped by DEM, large urban municipal centers | one from active conducted survey data uncertainty, two from risk map uncertainty, and adjusted by population and urbanization | |  |
| 38 | The burden of malaria mortality among African children in the year 2000 | Systematic review and Poisson regression models | childhood malaria mortality | | literature | | parasite prevalence: used as a proxy for malaria transmission intensity. urban vs. rural residence, health infrastructure, study year, geographical characteristics | explored the robustness of estimates under varying assumptions about deaths with unknown causes, urban populations, and epidemic deaths. | |  |
| 47 | Burden of symptomatic dengue infection in children at primary school in Thailand: a prospective study | DALYs equation | children febrile status, whether acute dengue, severity grade | | cohort | | epidemiological laboratory-confirmed dengue (DF, DHF), non-dengue febrile illnesses, duration of illness DALY-specific disability weight (0.81), age-weighting parameters, discount rate, demographic Pediatric population, age stratification, healthcare system private clinics, public hospitals, health-seeking behavior, costs (treatment, medication, income loss), economic assumptions, healthcare expenditure estimates | Empirical variability summarized using interquartile ranges (IQRs) from mortality and transmission studies; uncertainty propagated through categorical risk-class reassignment (urban vs rural) and recalculation of populations at risk; no formal probabilistic uncertainty propagation or Monte Carlo simulation; results reported as median estimates with IQR bounds. | |  |
| 48 | Disability adjusted life years lost to dengue in Brazil | a set of expansion factors under Monte Carlo method | official reported case number | | ministry of health, official report | | outcomes (cure or death), laboratory-confirmed diagnoses demographic age of onset, population size, years of life lost, DALY-Specific Disability grade, social discount rate, age-weighting parameters methodological time period (multi-year average), global burden of disease parameters | Monte Carlo simulation (1,000 iterations) with probabilistic distributions assigned to DALY parameters (age of onset, duration, years of life lost); sensitivity analysis using a range of multiplication factors to account for under- and over-reporting of dengue cases; results reported as ranges around annual DALY estimates. | |  |
| 84 | Cost and disease burden of dengue in Cambodia | DALYs calculation | dengue incidence and case fatality rates | | active community-based surveillance | | country | Deterministic DALY calculation using age-specific incidence and mortality from active surveillance; sensitivity analysis varying health-seeking behavior assumptions and discounting of premature mortality; no probabilistic uncertainty propagation or Monte Carlo simulation for DALYs. | |  |
| 39 | Estimating the global clinical burden of Plasmodium falciparum malaria in 2007. | Bayesian geostatistical model, Bayesian nonparametric model | Case number, population, parasite prevalence | | national report, literature | | parasite prevalence, clinical incidence, transmission intensity, temporal volatility, population density, urban and rural classification, regional diffs in vector ecology, drug resistance, co-infection with p vivax, genetic factors, underreporting correction factors, treatment-seeking behaviour, diagnostic accuracy, | Combination of two models uncertainty in a fully Bayesian framework, Incorporated through 250,000 model realizations to account for uncertainty in both endemicity and incidence estimates. | |  |
| 49 | Dengue in Thailand and Cambodia: an assessment of the degree of underrecognized disease burden based on reported cases. | multiplication factors (MFs) | cohort data and dengue reporting data | | cohort study and national surveillance | | demographic age stratification (0–4, 5–9, 10–14 years), geographic provincial-level data (Kamphaeng Phet, Ratchaburi), national-level aggregation methodological annual data (2003–2007), adjustments for missing data, age group assumptions | Underrecognition assessed using age-specific multiplication factors derived from comparisons of prospective laboratory-confirmed cohort incidence with provincial and national surveillance data; uncertainty reflected through ranges of multiplication factors across age groups and years; no formal probabilistic uncertainty propagation or Monte Carlo simulation. | |  |
| 12 | Worldwide incidence of malaria in 2009: estimates, time trends, and a critique of methods | method 1 adjust reported data; method 2 use risk map, model incidence | reported case, fever rate and seek health care rate estimate, incidence rate | | WHO, MARA project, DHS, MICS, literature review | | method 1 using reporting completeness, the proportion of suspected malaria cases that is parasite-positive, the proportion of malaria cases that is due to each Plasmodium species, the extent to which patients seek treatment, and whether patients use public sector health facilities; method 2 using climate suitability. | An underlying distribution was assumed for each of the parameters used in incidence estimation. Palisade@Risk (version 5.0) was used to sample from the distributions assumed for each parameter and each country. Latin Hypercube sampling without replacement was carried out using a pseudorandom number generator (Mersenne twister). For each country, we performed 1,000 calculations to yield a plausible distribution for the annual incidence of malaria cases, summarized with the mean, and bounded by 5th and 95th centiles. | |  |
| 75 | Leishmaniasis worldwide and global estimates of Its Incidence | expansion factor | reported data, published data, expansion factor from literature, expert opinion | | literature, official report, expert opinion from questionnaire | | access to antileishmanial medicines, treatment-seeking behaviour, drug procurement, health system characteristics, population data, basic social and health data, reservoir characteristics, vector control | Incidence estimated as ranges using reported national surveillance data adjusted by underreporting multipliers derived from published empirical studies, literature review, and expert judgment; uncertainty expressed as country- and region-specific incidence ranges rather than confidence intervals; no probabilistic uncertainty propagation or simulation. | |  |
| 40 | Spatially explicit burden estimates of malaria in Tanzania: bayesian geostatistical modeling of the malaria indicator survey data. | Bayesian geostatistical models with Markov chain Monte Carlo (MCMC) simulation, Bayesian kriging | prevalence | | survey | | land surface temperature (LST), rainfall, normalized difference vegetation index (NDVI), altitude and distance to nearest permanent water body | Markov chain Monte Carlo (MCMC) simulation | |  |
| 50 | Economic and disease burden of dengue in Southeast Asia. | expansion factor to adjust | case data | | surveillance data, WHO, and published studies | | epidemiological reported cases, underreporting adjustments (EFs), symptomatic episodes, surveillance variables healthcare hospitalization rates, ambulatory care rates, duration of illness demographic age distribution of fatal cases, population data, regional adjustments methodological disability weights (0.81), duration of illness (hospitalized and ambulatory), DALY components (YLL and YLD) | Monte Carlo simulations | |  |
| 17 | Use of expansion factors to estimate the burden of dengue in Southeast Asia: a systematic analysis. | expansion factor to adjust | reported case and death number | | literature | | health-quality index | 20,000 Monte Carlo simulations | |  |
| 79 | The global distribution and burden of dengue. | 336 BRT, and a hierarchical Bayesian model linked the cohort incidence data with probability of occurrence from BRT | geo-located occurrence records, incidence data, population surfaces | | literature, online platforms e.g. HealthMap | | annual maximum and minimum precipitation, temperature, average NDVI, categorical demarcations of urban and peri-urban areas; an urban accessibility metric defining the travel time to nearest city of 50,000 people or more by land- or water-based travel; and an indicator of relative poverty, human movement between urban centres | BRT ensemble with 336 model runs for risk map uncertainty, Bayesian MCMC posterior distribution for 95% credible intervals. The sensitivity analysis was performed using different combinations of pseudo-data generating parameters np, na and μ. | |  |
| 89 | Estimating leptospirosis incidence using hospital-based surveillance and a population-based health care utilization survey in Tanzania | multiplier methods | survey data based on population and fever surveillance based on hospital | | survey and hospital surveillance | | diagnostic test sensitivity/specificity, MAT results on paired sera healthcare system health-seeking preferences, referral adjustments, fever surveillance coverage demographic population by age group (0–4, 5–14, ≥15 years), census data urban and rural, methodological multipliers (KCMC, MRH, enrolment, time, paired sera, MAT sensitivity), referral adjustment | Deterministic multiplier-based incidence estimation using hospital surveillance data combined with population-based health care utilization survey; sensitivity assessed by alternative health-care–seeking assumptions, diagnostic sensitivity/specificity adjustments, and inclusion of confirmed versus confirmed + probable cases; uncertainty reported as incidence ranges rather than probabilistic intervals. | |  |
| 41 | A novel approach for measuring the burden of uncomplicated Plasmodium falciparum malaria: application to data from Zambia. | Novel approach based on point prevalence of malaria attributable disease | Survey data, fever patterns in malaria therapy patients, data on recall bias | | literature, national survey and national report | | fever patterns, malaria therapy data, recall bias | Bayesian analysis, interval estimates, comparison with existing estimates, acknowledgment of assumptions and potential biases. | |  |
| 61 | Yellow fever in Africa: estimating the burden of disease and impact of mass vaccination from outbreak and serological data. | GLM | records of case，locations of yellow fever outbreaks, serological surveys data, vaccination coverage data | | Weekly Epidemiological Record (WER), the WHO disease outbreak news (DON), an internal WHO database, and the published literature | | rainfall, day- and night-time air temperatures, land cover classifications, the enhanced vegetation index, the middle infrared reflectance, longitude, latitude, and altitude and surveillance quality indicators, vaccination coverage | fitted beta distribution to the point estimates and 95% credibility intervals of the proportion of cases among infections and the case fatality ratio and generated samples from both distributions that we then multiplied by the number of infections estimated during each MCMC iteration. | |  |
| 51 | Economic and disease burden of dengue illness in India | adjust by expansion factor | numbers of hospitalized clinically diagnosed dengue, number of beds | survey, expert and surveillance | | diagnostic testing rates (IgG, IgM, NS1), annual case variability healthcare system facility types (public/private, small/medium/large hospitals), ambulatory vs. hospitalized cases, bed capacity demographic district-level data, state and national population, regional stratification Methodological Adjustment factors (Madurai study, state, and national level), expert opinion, surveillance data | | To calculate Cis, we used the probabilistic sensitivity analysis with triangular distributions for 1) the share of cases treated in the hospital sector, 2) the adjustment factor, 3) the share treated in the private sector, 4) cost per day in private hospitals, 5) cost per day in public hospitals, 6) length of stay in private hospitals, and 7) length of stay in public hospitals. Available data for each parameter generated the minimum, maximum and best estimate. For each sensitivity analysis, we performed 1,000 Monte Carlo iterations with independent drawings for each parameter. We presented results as mean values and 95% CIs in a tornado diagram. |  |  |
| 42 | Estimates of the changing age-burden of Plasmodium falciparum malaria disease in sub-Saharan Africa | a transmission mathematical model incorporating acquisition and loss of immunity under a Bayesian framework using Markov Chain Monte Carlo (MCMC) | parasite prevalence or entomological inoculation rate, EIR and incidence of disease | | literature | | not applicable | Bayesian MCMC | |  |
| 74 | Global morbidity and mortality of leptospirosis: A systematic review. | Linear regression and Monte Carlo modelling | published morbidity and mortality (age and sex-statified cases, country-specific crude incidence, ratio of clinically suspected: laboratory-comfimed cases, age and sex stratified deaths, country-specific crude incidence, case fatality, ratio of cliniclly suspected: labboratory-confirmed deaths) | | literature | | whether the country is a tropical island, precent urbanization of the population, distance from the equator in degrees latitude, the mean years of life expectancy at birth | Systematic review with study quality assessment; multivariable regression modelling to predict country-level incidence where data were sparse; Monte Carlo simulation to propagate uncertainty from incidence, case fatality ratios, age–sex risk distributions, and underreporting due to incomplete diagnostic testing; results reported with 95% confidence intervals for regional and global estimates | |  |
| 52 | Economic and disease burden of dengue in Mexico | expansion factor | official reported case number | | survey and ministry of health | | healthcare-seeking behavior, hospitalization, healthcare system quality, age distribution, population-level adjustments, environmental factors: local factors impacting dengue transmission, such as seasonal variability in cases. socio-cultural factors: awareness and participation in dengue surveillance. socio-cultural norms influencing healthcare-seeking behaviour. disability weights | We used a probabilistic sensitivity analysis to address the uncertainty in our estimates of the disease and economic burden of dengue. We computed 10,000 Monte Carlo simulations simultaneously varying our parameter estimates for EFs, unit costs, days lost per episode, health service utilization, and household impact using RiskAMP (iterations drew random from the distribution of each input using the Mersenne Twister random number generator). | |  |
| 53 | Economic cost and burden of dengue in the Philippines | adjust by expansion factor | dengue incidence, national case and death data from | | expert, Philippines' national and regional dengue surveillance systems, cohort and DOH National Epidemiology Center | | healthcare system setting (ambulatory vs. hospitalized), sector (public vs. private), treatment and testing rates demographic cohort data, population distribution, allocation of unknown cases Methodological Adjustment factors, outlier detection, cost estimation methods | Results are presented as mean values with 95% CIs. For each sensitivity analysis, we performed 1,000 Monte Carlo simulations with an associated triangular distribution. Results are presented as mean values and 95% CIs. | |  |
| 76 | Predicted global distribution of Burkholderia pseudomallei and burden of melioidosis | BRT, negative binomial, multivariable logistic regression | human cases, animal cases and presence of B.pseudomallei; incidence, case fatality rate | | literature, case reports, and websites | | soil type, precipitation, temperature, vegetation/moisture index; prevalence of diabetes, prevalence of aboriginal population; national-level healthcare expenditure, national-level under 5 mortality rate | bootstrap resampling procedure to randomly select data sets of both positive occurrences and pseudo-negative locations | |  |
| 14 | Mapping plasmodium falciparum mortality in Africa between 1990 and 2015. | ensemble of independently developed microsimulation models | clinical incidence, database of studies of parasite rate | | Malaria Atlas Project | | environmental covariates to generate parasite rate map, (bed-net distribution, relative drug use, antimalarial coverage, antimalarial drug efficacy) to get the effective treatment coverage | Spatiotemporal geostatistical modelling integrating multiple data sources; uncertainty propagated through each modelling step (incidence, treatment coverage, drug efficacy, and case fatality) using simulation analysis; final outputs reported with 95% uncertainty intervals at 5-km grid, national, and regional levels, with CoDCorrect applied to align cause-specific mortality with all-cause mortality. | |  |
| 81 | Comparison of the estimated incidence of acute leptospirosis in the Kilimanjaro region of Tanzania between 2007–08 and 2012–14 | incidence estimation using multipliter | serological survey data, population denominator | | hospital surveillance, laboratory, healthcare utilization survey, census | | adjustment multipliers: healthcare-seeking behaviour multipliers, enrolment rate multipliers, blood draw multipliers, diagnostic test sensitivity/specificity, paired-unpaired sera, time, study duration | alternative healthcare utilization multipliers, diagnostic sensitivity scenarios | |  |
| 43 | Fine-scale mapping by spatial risk distribution modeling for regional malaria endemicity and its implications under the low-to-moderate transmission setting in western Cambodia. | GLM (We first used the maximum likelihood method to examine the predictor variables and then, based on the results, we used the MCMC method in the Bayesian modeling frame to estimate the uncertainty about the relationships represented by α, β, and γ) | case data | | Cambodia Malaria Bulletin report | | NDVI, NDWI, LSWI, digital elevation, p. Falciparum temperature suitability index, population density, sufficient ownership of LLIN, treatment failure rate by artemisinin combination therapy. | Used the MCMC method in the Bayesian modelling frame to estimate the uncertainty about the relationships represented by α, β, and γ. | |  |
| 55 | Indonesian dengue burden estimates: review of evidence by an expert panel. | an expert Delphi panel and expansion factor | reported dengue cases | | surveillance | | not applicable | Voting summaries from the final round, and median estimates from bootstrapping resampling and their 95% CIs. | |  |
| 56 | Disease burden of dengue in the Philippines: adjusting for underreporting by comparing active and passive dengue surveillance in Punta Princesa, Cebu City. | expansion factor and Delphi Panel Workshop | active surveillance of symptomatic DENV infections and passive surveillance data | | surveillance and one survey | | duration of illness, age distribution, case fatality rates, proportion of cases hospitalized, healthcare utilization, length of hospital stay, disability weight, reporting rates, age-weights and time-discounting | Computed 10,000 Monte Carlo simulations for each parameter, To estimate uncertainty for nonfatal EF, we obtained the standard deviation from the sample of monthly estimates of reporting rates and assumed a truncated normal distribution (censored at 5%). | |  |
| 44 | Incidence and admission rates for severe malaria and their impact on mortality in Africa. | Simulation-based model | national or geographic reported clinical and treatment data, coverage of treatment, in-patient deaths | | The World Health Organization’s annual World Malaria Report | | demographics, malaria prevalence, treatment coverage | Simulation-based modelling using OpenMalaria with country-specific transmission, treatment coverage, and case fatality assumptions; alternative estimation approaches (prediction-biased vs deaths-adjusted) used to bracket uncertainty in severe malaria incidence and admission rates; sensitivity analyses conducted on key parameters (notably community vs in-patient case fatality odds ratios); uncertainty expressed through ranges and comparative scenarios rather than formal probabilistic intervals. | |  |
| 62 | Existing and potential infection risk zones of yellow fever worldwide: a modelling analysis | Poisson point process boosted regression tree model for risk map, GLM to estimate the calibration factors | locations where at least one laboratory-confirmed symptomatic human infection of yellow fever virus had been reported in any given year | | online sources, peer-reviewed literature, and WHO reports | | evergreen broadleaf forest, urban and built up, and cropland mosaics land cover classes (proportional), elevation, tasselled cap wetness/a measure of surface moisture, enhanced vegetation index, Aedes aegypti temperature suitability index, A. aegypti habitat suitability, predictive species distribution of suspected reservoir non-human primates | An ensemble of 100 Bernoulli boosted regression tree models (sub-models), Each sub-model was trained to a separate bootstrap dataset randomly sampled with replacement from the complete occurrence/background dataset for that continent. | |  |
| 57 | Estimating dengue incidence and hospitalization in Malaysia, 2001 to 2013. | incidence-prevalence-mortality (IPM) model | Data on dengue prevalence and dengue hospitalization data | | survey, national surveillance and Health Informatic Centre (HIC) of the Ministry of Health | | not applicable | Incidence–prevalence–mortality (IPM) model combining sero-prevalence surveys and dengue-specific mortality; sensitivity analysis varying post-2008 rural sero-prevalence (±20%) to assess impact on incidence trends; Poisson regression with generalized estimating equations to estimate hospitalization volumes for hospitals with missing data; uncertainty reflected through scenario ranges and trend confidence intervals rather than full probabilistic uncertainty propagation. | |  |
| 18 | Nationally-representative serostudy of dengue in Bangladesh allows generalizable disease burden estimates. | Bayesian framework with a Matern spatial correlation structure using integrated nested Laplace approximations, catalytic model to estimate forc of infection | seropositivity | | survey | | population size, age category and sex | Nationally representative serosurvey with survey-weighted analyses; Bayesian hierarchical logistic regression with household- and community-level random effects and spatial (Matérn) covariance to quantify spatial uncertainty; posterior predictive simulations to generate 95% credible intervals for seroprevalence and spatial risk maps; sensitivity analyses on assumptions about duration of transmission and sampling design (number of communities and individuals); catalytic models with uncertainty bounds used to estimate force of infection and annual infections. | |  |
| 13 | Mapping the global prevalence, incidence, and mortality of Plasmodium falciparum, 2000-17: a spatial and temporal modelling study. | Prevalence to incidence conversion; Bayesian spatiotemporal geostatistical model; Gaussian process regression; Generalized additive mixed models (GAMM);Treatment-seeking model;time-series modelling;machine learning; traditional cause of death ensemble model (CODEm) | P falciparum parasite rate in Africa and reported P falciparum malaria cases for the rest 70 countries | | survey, literature, national surveillance | | IGBP combined forest, EVI mean, EVI SD, LST daytime annual mean, LST daytime annual SD, LST night annual mean, LST night annual SD, TCB annual mean, TCB annual SD, precipitation, accessibility, nighttime light, elevation, CGIAR-CSI global PET database, population, Plasmodium falciparum parasite rate, treatment-seeking behaviours, gross domestic product (GDP) growth, health expenditure (% of GDP) and out-of-pocket health expenditure (% of total expenditure on health), rates of primary education completion | Bayesian spatiotemporal geostatistical modelling with posterior simulation; uncertainty propagated from parasite prevalence surveys, routine surveillance data, intervention coverage, and cause-of-death data through ensemble and ecological downscaling models; stochastic posterior draws used to generate 95% uncertainty intervals for pixel-level, subnational, national, regional, and global estimates, with final mortality constrained to all-cause mortality envelopes. | |  |
| 45 | Mapping the global endemicity and clinical burden of Plasmodium vivax, 2000-17: a spatial and temporal modelling study. | Bayesian geospatial models; time-series models for outside Africa; model between clinical incidence and p vivax parasite rate | routine malaria metric surveillance data; household survey; parasite rate | | subnational surveillance and Malaria Atlas Project | | IGBP combined forest, EVI mean, EVI SD, LST daytime annual mean, LST daytime annual SD, LST night annual mean, LST night annual SD, TCB annual mean, TCB annual SD, precipitation, accessibility, nighttime light, elevation, CGIAR-CSI global PET database, population, Plasodium falciparum parasite rate, treatment-seeking behaviours, gross domestic product (GDP) growth, health expenditure (% of GDP) and out-of-pocket health expenditure (% of total expenditure on health),rates of primary education completion | Bootstrapping was used to obtain 100 samples that characterised the uncertainty in the model, summarised as pixel-level SD estimates and upper or lower incidence bounds | |  |
| 58 | Estimating the burden of dengue and the impact of release of wMel Wolbachia-infected mosquitoes in Indonesia: a modelling study. | BRT and GBM | occurrence, incidence and seroprevalence of dengue | | literature | | GDP, annual cumulative precipitation, minimum annual relative humidity, mosquito suitability, urban/rural status, temperature suitability | Multi-stage ensemble modelling combining heterogeneous burden estimates, spatial risk models, and transmission dynamics; uncertainty propagated using bootstrapped boosted regression trees, Bayesian ensemble sampling, and multiple parameterisations of dengue transmission models; Monte Carlo simulation used to generate uncertainty intervals for national burden, spatial burden maps, DALYs, and projected intervention impact, reported as 95% uncertainty intervals. | |  |
| 72 | Estimates of the global burden of Japanese encephalitis and the impact of vaccination from 2000-2015. | mathematical modelling method (catalytic model) in a Bayesian framework, catalytic model | age-stratified case data of 15 countries from systematic review | | literature and national report | | case, population, vaccinated population | - | |  |
| 92 | The effect of climate change on yellow fever disease burden in Africa. | GLM within Bayesian hierarchical model, temperature suitability model (mechanistic, Ross-Macdonald-based), serology-informed force of infection estimation | case report, occurrence reports, serological survey data, vaccination coverage estimates | | WHO database and published literature | | temperature range, temperature suitability and precipitation, longitude, population size, surveillance quality indicator | Full Bayesian uncertainty propagation, Posterior distributions reported with 95% credible intervals, Monte Carlo sampling used throughout modelling chain, Uncertainty in epidemiological parameters propagated, but climate model uncertainty not fully included. | |  |
| 65 | Estimating the burden of arboviral diseases in Colombia between 2013 and 2016. | expansion factor adjusted incidence+ corrected mortality and corrected by group, using correction for the proportion of deaths with death certificates, the Bennet–Horiuchi method and expansion factors | reported case database of dengue, chikungunya, and Zika, mortality records, population at risk, published parameters for chronic sequelae incidence, duration and disability weights | | national surveillance database, published literature | | age; Sex; disease severity (outpatient, hospitalized, severe); acute vs chronic manifestations; population at risk (altitude <2100 m, vector presence); expansion factors for underreporting; disease duration (acute and chronic phases); disability weights (disease- and phase-specific); mortality correction parameters (death registration completeness) | Deterministic sensitivity analysis with three scenarios: Conservative; Medium; Extreme; Scenario variation applied to: Expansion factors, Disease duration, Disability weights, Incidence of chronic sequelae, Mortality parameters, Results reported as ranges of DALYs across scenarios | |  |
| 59 | Mapping global variation in dengue transmission intensity. | random forest model | FOI data point | | literature | | precipitation, diurnal temperature, nocturnal temperature, enhanced vegetation index, middle infrared reflectance, altitude, population density, and per capita human birth rate | Machine-learning–based geospatial modelling of dengue force of infection using random forests fitted to serology- and surveillance-derived FOI estimates; spatial block bootstrapping and expectation–maximization downscaling used to quantify prediction uncertainty; 200 geographically stratified bootstrap replicates used to generate pixel-level means and 95% confidence intervals for FOI, burden estimates, reproduction numbers, and projected intervention impacts. | |  |
| 94 | Burden is in the eye of the beholder: Sensitivity of yellow fever disease burden estimates to modeling assumptions | estimate FOI with serology, estimate underreporting, project FOI everywhere, regress FOI against spatial covariates, estimate regression model weights, ensemble projection of FOI and deaths | serological surveys data, outbreak reports, population data, vaccination coverage | | literature, WHO records, UN | | NDVI, precipitation, temperature, elevation, longitude, latitude, travel time to nearest urban center, occurrence probability, richness of NHP species, frontier land cover, tropical ecotype, forests loss, health access quality index for regression of FOI | Bayesian posterior distributions, Dirichlet-multinomial models for underreporting estimation, ensemble modelling with cross validation, regression model uncertainty incorporated via stacked generalization, partitioning of variance according to sources (quantifies the relative contribution of spatial heterogeneity, serology scenario, regression model, statistical uncertainty) | |  |
| 64 | The global burden of yellow fever. | GLM MCMC under Bayesian framework | occurrence data, serological survey data | | literature | | annual temperature maximum, minimum, and range; population size; annual precipitation max, min and mean; enhanced vegetation index max, min, range and mean; middle infrared reflectance max, min, and mean; proportion of land cover; occurrence of all NHP families; occurrence of Ae.aegypti and Ae. albopictus; mean altitude; temperature suitability index for Ae. aegypti | We propagate uncertainty from both the parameter estimation and model structure. This is done through sampling proportionally from the posterior distributions of all 20 of the best-fitting models to produce 500 force of infection and thus burden predictions. | |  |
| 82 | Incidence estimates of acute q fever and spotted fever group rickettsioses, Kilimanjaro, Tanzania, from 2007 to 2008 and from 2012 to 2014 | multipliers | Hospital-based sentinel surveillance data combined with population-based healthcare utilization surveys | | sentinel surveillance and surveys | | age, healthcare-seeking behavior, diagnostic test sensitivity and specificity, time of enrollment, enrollment completeness | Uncertainty evaluated through sensitivity analysis using alternative survey scenarios for healthcare-seeking behaviors and ranges in diagnostic test performance. 95% confidence intervals were applied to prevalence and healthcare utilization data to produce uncertainty ranges. | |  |
| 100 | Mapping the distributions of mosquitoes and mosquito-borne arboviruses in China. | Two-stage generalized boosted regression tree (GBRT) | presence of the virus at county (any record of positive detection from human case, host animals or mosquitoes) and reported incidence | | the Chinese Scientific Data Center for Public Health, literature review, GenBank | | environmental (basin, paddy field, rainfed cropland, forest, grasslands, river, rural residential land, other construction land); ecoclimatic(annual mean temperauter, isothermality, temperature seasonality, mean temperature of wettest quarter, mean temperature of warmest quarter, annual cumulative precipitation, precipitation seasonality, precipitation of driest quarter);social(index of case importation, proportion of women, propotion of >= 60 years old, number of gengral hospitals, number of clinics); biological(density of population, mammalian richness, density of pig, density of cattle, density of duck, goat, sheep, chicken, presence of mosquitoes) | Ensemble machine-learning modelling using boosted regression trees (BRT) and generalized BRT with repeated bootstrapping; uncertainty quantified via distributions of relative contributions, receiver operating characteristic (ROC) metrics (AUC and partial AUC), and 2.5–97.5% percentiles across 100 bootstrap models; model-predicted presence probabilities averaged across ensembles to reflect uncertainty in mosquito and arbovirus distributions. | |  |
| 46 | Improving disaggregation models of malaria incidence by ensembling non-linear models of prevalence. | machine learning models (elastic net, Random Forest, projection pursuit regression, neural networks and boosted regression trees (gradient boosted models, subsequently GBM));disaggregation regression model | binomial prevalence data, incidence (aggregated to administrative units) | | Malaria Atlas Project prevalence survey database (publicly available), government reports | | annual mean and log standard deviation of land surface temperature, enhanced vegetation index, malaria parasite temperature suitability index, elevation, tasseled cap wetness, log accessibility to cities and log night lights | Two-stage modelling framework combining machine-learning predictions and Bayesian disaggregation regression; uncertainty captured through spatial Gaussian random fields (Matérn covariance), polygon-level iid random effects, and penalised complexity priors; model performance and robustness evaluated using random and spatial cross-validation, with uncertainty assessed via correlation metrics and coverage of 80% credible intervals rather than full posterior uncertainty maps. | |  |
| 80 | Mapping schistosomiasis risk in Southeast Asia: a systematic review and geospatial analysis | Bayesian geostatistical model | survey site prevalence data | | literature and Global Atlas of Helminth infection (GAHI) | | distance to water bodies, annual precipitation, land surface temperature (daytime), NDVI (vegetation index), GDP, infant mortality rate (IMR), urban/rural classification, proportion with improved sanitation and water access. | Bayesian credible intervals (CrI); Cross-validation of predictive accuracy; Posterior parameter distributions for spatial range (r ≈ 48 km) and marginal variance (σ² ≈ 8.4); Validation r = 0.79–0.81 (P < 0.01). | |  |
| 70 | Estimating the seroincidence of scrub typhus using antibody dynamics after infection | Bayesian hierarchical models | longitudinal antibody data and population serosurveys data | | cohort survey and population serosurveys | | multiple countries | Bayesian credible intervals MCMC sampling and likelihood-based CIs, incorporated measurement and biologic noise in the sero-response model | |  |
| 66 | The global health and economic burden of chikungunya from 2011 to 2020: a model-driven analysis on the impact of an emerging vector-borne disease | DALYs computation with country-specific under-reporting adjustment, Monte Carlo simulation | case data | | PAHO/PLISA, ProMED, ECDC, CDC, national MoHs (Brazil, Indonesia, Philippines, Singapore), literature, national survey/report, | | chronicity rate, case fatality, disease duration, disability weight, under reporting factor, GDP PPP, labour force participation | Monte Carlo simulation 5000 iterations | |  |
| 95 | Estimating the health effects of COVID-19-related immunisation disruptions in 112 countries during 2020-30: a modelling study | modelling groups from the Vaccine Impact Modelling Consortium (VIMC), for yellow fever, UND yellow fever model: estimate FOI with serology, estimate underreporting, project FOI everywhere, regress FOI against spatial covariates, estimate regression model weights, ensemble projection of FOI and deaths; Imperial yellow fever model: stochastic, dynamic transmission model, using Bayesian framework to estimate FOI and basic reproduction number of human-to-human transmission. | demography data | | public database UNWPP | | 18 spatial covariates, demography, vaccination | Two models for each disease to include structural uncertainty, 200 estimates using different sample of input parameters taken from distributions, CIs were calculated by combining the full probabilistic distributions of effect | |  |
| 77 | The public health impact and cost-effectiveness of the R21/Matrix-M malaria vaccine: a mathematical modelling study | Semi-mechanistic immunological model linking anti-circumsporozoite protein (CSP) antibody titres to vaccine efficacy; Age-structured, individual-based malaria transmission model (based on malariasimulation package);Cost-effectiveness model incorporating DALYs and program costs. | antibody titers and clinical episodes, trial data, demographic and transmission data | | trial dataset, public website | | parasite prevalence (PfPR₂–₁₀) as main epidemiological driver; Seasonality pattern (perennial vs seasonal); Age and immunity (maternal + acquired); Treatment access (45 % treated); Vaccination coverage & booster adherence. | Bayesian framework (MCMC) for antibody efficacy model (posterior medians & 95 % CrI); 50 parameter draws from antibody transmission model for stochastic uncertainty; Sensitivity analyses: vaccine price, delivery costs, case management costs. | |  |
| 67 | Global, regional and national burden of chikungunya: force of infection mapping and spatial modelling study | Random forest model to predict force of infection, catalytic model and chikungunya prognosis model | force of infection data, duration of illness, disability weights, life expectancies, disease progression probabilities | | previous publication, public website/database | | temperature suitability for dengue virus transmission, annual precipitation, GDP per capita, probabilities of Aedes aegypti and Aedes albopictus presence, and environmental suitability for chikungunya. | 100 random-forest iterations; Spatial block cross-validation; Latin Hypercube Sampling (1000 runs) for DALY uncertainty; 95 % uncertainty intervals (UIs) for all metrics. | |  |
| 85 | Global estimation of dengue disability weights based on clinical manifestations data | meta-analysis and Monte Carlo simulation model | clinical manifestations of dengue | | literature | | age group, region, year, clinical manifestations | Monte Carlo simulation (5000 iterations) with probabilistic sampling used to produce uncertainty intervals (UI), beta distribution for symptom frequency | |  |
| 68 | Global burden of chikungunya virus infections and the potential benefit of vaccination campaigns | logistic regression for probability of outbreaks, serocatalytic model MCMC/SMC based, SIRV model | seroprevalence, case report, mosquito distribution maps, population data | | literature, ministry report, public website | | Aedes aegypti and A. albopictus probability of presence (mosquito suitability); Human population density (weighted by mosquito presence); Age structure (12 groups) and sex distribution; Healthcare Access & Quality Index (for surveillance strength); Baseline immunity by region; Seasonal transmission coefficients (β(t)). | Bayesian MCMC and SMC for FOI and serocatalytic model posterior quantiles, bootstrapped 1000 simulations for vaccine impact and DALY output, sensitivity analyses on 23 parameters | |  |
| 69 | Modeling the impact of vaccine campaigns on the epidemic transmission dynamics of chikungunya virus outbreaks | Time-varying compartmental SIR (Susceptible–Infected–Removed) transmission model | weekly chikungunya case data, nationwide seroprevalence study | | national surveillance database, seroprevalence study | | age and sex, temperature, prior immunity | Binomial & negative binomial likelihoods to generate uncertainty of seroprevalence estimates which was propagated to infection number, MCMC posterior distributions 4 chains and 25000 iterations | |  |
| 78 | Vaccination strategies, public health impact and cost-effectiveness of dengue vaccine TAK-003: A modeling case study in Thailand | dynamic deterministic compartmental transmission model, expansion factor to adjust underreporting | epidemiological data, vaccine efficacy, population, economic inputs | | ministry, literature | | infection risk, seasonality, serostatus, transmission probability per bite, hospitalization and symptomatic probability, cost | 70 forward simulations, scenario analyses, 95% confidence/credible intervals | |  |
| 71 | Estimating scrub typhus and murine typhus incidence among adolescents and adults in Yangon, Myanmar | multiplier methods | demographics, socioeconomic  status, and the healthcare-seeking behaviour, causes of community-onset febrile illness | | sentinel hospital surveillance | | healthcare seeking behaviour, referral bias, diagnostic test sensitivity/specificity, enrolment rate, weekday-only enrolment (time multiplier) | One-way sensitivity analysis using 95% CI bounds of multipliers; Diagnostic sensitivity: 54% (scrub typhus), 97.5% (murine typhus); Wide range for healthcare utilization multiplier (62–12,444); Uncertainty propagated via upper/lower multiplier bounds. | |  |
