# Supplementary material for: Methods for estimating the burden of acute tropical infectious diseases: A scoping review
Source: PLoS Negl Trop Dis. 2026 May 4;20(5):e0013359. doi: 10.1371/journal.pntd.0013359 (PMC13160447; doi:10.1371/journal.pntd.0013359)
Supplement: S5 Table — (DOCX) [file pntd.0013359.s005.docx]

**S5 Table. List of articles excluded at full-text review stage and corresponding justification based on predefined eligibility criteria.**

| **Reason for Exclusion** | **Articles** |
| --- | --- |
| Forecast incidence based on reported data, not burden estimation | Nguyen, Van-Hau et al. “Deep learning models for forecasting dengue fever based on climate data in Vietnam.” PLoS neglected tropical diseases vol. 16,6 e0010509. 13 Jun. 2022, doi:10.1371/journal.pntd.0010509 |
| Generated risk map, not direct burden result | Che, Tian-Le et al. “Mapping the risk distribution of Borrelia burgdorferi sensu lato in China from 1986 to 2020: a geospatial modelling analysis.” Emerging microbes & infections vol. 11,1 (2022): 1215-1226. doi:10.1080/22221751.2022.2065930 |
| Not develop, adapt, or apply a new burden estimation framework, data extracted from GBD | Yang, Xiaorong et al. “Global burden for dengue and the evolving pattern in the past 30 years.” Journal of travel medicine vol. 28,8 (2021): taab146. doi:10.1093/jtm/taab146 |
| Lyme disease not acute fever | Mac, Stephen et al. “Estimating the population health burden of Lyme disease in Ontario, Canada: a microsimulation modelling approach.” CMAJ open vol. 9,4 E1005-E1012. 16 Nov. 2021, doi:10.9778/cmajo.20210024 |
| Forecast study, not burden estimation | Karunaweera, Nadira D et al. “Spatiotemporal distribution of cutaneous leishmaniasis in Sri Lanka and future case burden estimates.” PLoS neglected tropical diseases vol. 15,4 e0009346. 23 Apr. 2021, doi:10.1371/journal.pntd.0009346 |
| Not acute febrile infectious disease | Vinkeles Melchers, Natalie V S et al. “The burden of skin disease and eye disease due to onchocerciasis in countries formerly under the African Programme for Onchocerciasis Control mandate for 1990, 2020, and 2030.” PLoS neglected tropical diseases vol. 15,7 e0009604. 26 Jul. 2021, doi:10.1371/journal.pntd.0009604 |
| Generated the vulnerability, not direct burden result | Zafar, Sumaira et al. “Development and Comparison of Dengue Vulnerability Indices Using GIS-Based Multi-Criteria Decision Analysis in Lao PDR and Thailand.” International journal of environmental research and public health vol. 18,17 9421. 6 Sep. 2021, doi:10.3390/ijerph18179421 |
| Predicted suitable habitats, not direct burden result | Zhao, Guo-Ping et al. “Mapping ticks and tick-borne pathogens in China.” Nature communications vol. 12,1 1075. 17 Feb. 2021, doi:10.1038/s41467-021-21375-1 |
| Reported burden, and no model for burden estimation | Wangrangsimakul, Tri et al. “The estimated burden of scrub typhus in Thailand from national surveillance data (2003-2018).” PLoS neglected tropical diseases vol. 14,4 e0008233. 14 Apr. 2020, doi:10.1371/journal.pntd.0008233 |
| Lymphatic Filariasis not acute febrile infectious disease | Local Burden of Disease 2019 Neglected Tropical Diseases Collaborators. “The global distribution of lymphatic filariasis, 2000-18: a geospatial analysis.” The Lancet. Global health vol. 8,9 (2020): e1186-e1194. doi:10.1016/S2214-109X(20)30286-2 |
| Predicted the vulnerability, not direct burden result | Pham, Nga T T et al. “Assessing and modelling vulnerability to dengue in the Mekong Delta of Vietnam by geospatial and time-series approaches.” Environmental research vol. 186 (2020): 109545. doi:10.1016/j.envres.2020.109545 |
| Model but not significant association between incidences in human with incidence in livestock | Charypkhan, Duriya et al. “Economic and health burden of brucellosis in Kazakhstan.” Zoonoses and public health vol. 66,5 (2019): 487-494. doi:10.1111/zph.12582 |
| Reported case data and used WHO DALY calculation method | Swain, Subhashisa et al. “Distribution of and associated factors for dengue burden in the state of Odisha, India during 2010-2016.” Infectious diseases of poverty vol. 8,1 31. 6 May. 2019, doi:10.1186/s40249-019-0541-9 |
| Post-treatment lyme disease | DeLong, Allison et al. “Estimation of cumulative number of post-treatment Lyme disease cases in the US, 2016 and 2020.” BMC public health vol. 19,1 352. 24 Apr. 2019, doi:10.1186/s12889-019-6681-9 |
| Generated risk map, not direct burden result | Ding, Fangyu et al. “Risk factors and predicted distribution of visceral leishmaniasis in the Xinjiang Uygur Autonomous Region, China, 2005-2015.” Parasites & vectors vol. 12,1 528. 8 Nov. 2019, doi:10.1186/s13071-019-3778-z |
| Generated endemicity, not direct burden result | Kang, Su Yun et al. “Spatio-temporal mapping of Madagascar's Malaria Indicator Survey results to assess Plasmodium falciparum endemicity trends between 2011 and 2016.” BMC medicine vol. 16,1 71. 23 May. 2018, doi:10.1186/s12916-018-1060-4 |
| Predicted probability of presence, not direct burden result | Sun, Ruo-Xi et al. “Mapping the distribution of tick-borne encephalitis in mainland China.” Ticks and tick-borne diseases vol. 8,4 (2017): 631-639. doi:10.1016/j.ttbdis.2017.04.009 |
| No direct burden result, but malaria ecology index | McCord, Gordon C, and Jesse K Anttila-Hughes. “A Malaria Ecology Index Predicted Spatial and Temporal Variation of Malaria Burden and Efficacy of Antimalarial Interventions Based on African Serological Data.” The American journal of tropical medicine and hygiene vol. 96,3 (2017): 616-623. doi:10.4269/ajtmh.16-0602 |
| Analysis from GBD | Karimkhani, Chante et al. “Global burden of cutaneous leishmaniasis: a cross-sectional analysis from the Global Burden of Disease Study 2013.” The Lancet. Infectious diseases vol. 16,5 (2016): 584-591. doi:10.1016/S1473-3099(16)00003-7 |
| Generated force of infection and R0, not direct burden result | Imai, Natsuko et al. “Estimating Dengue Transmission Intensity from Case-Notification Data from Multiple Countries.” PLoS neglected tropical diseases vol. 10,7 e0004833. 11 Jul. 2016, doi:10.1371/journal.pntd.0004833 |
| It used DLMN to evaluate the association between three factors (NDVI, LST, precipitation) with malaria mortality and no burden estimate | Sewe, Maquins Odhiambo et al. “Remotely Sensed Environmental Conditions and Malaria Mortality in Three Malaria Endemic Regions in Western Kenya.” PloS one vol. 11,4 e0154204. 26 Apr. 2016, doi:10.1371/journal.pone.0154204 |
| Modelled the relative risk not direct burden result | Okami, Suguru, and Naohiko Kohtake. “Fine-Scale Mapping by Spatial Risk Distribution Modeling for Regional Malaria Endemicity and Its Implications under the Low-to-Moderate Transmission Setting in Western Cambodia.” PloS one vol. 11,7 e0158737. 14 Jul. 2016, doi:10.1371/journal.pone.0158737 |
| Generated risk map, not direct burden result | Alimi, Temitope O et al. “A multi-criteria decision analysis approach to assessing malaria risk in northern South America.” BMC public health vol. 16 221. 3 Mar. 2016, doi:10.1186/s12889-016-2902-7 |
| Review | Messina, Jane P et al. “The many projected futures of dengue.” Nature reviews. Microbiology vol. 13,4 (2015): 230-9. doi:10.1038/nrmicro3430 |
| Exploring relationship between incidence and prevalence, no estimated burden result | Cameron, Ewan et al. “Defining the relationship between infection prevalence and clinical incidence of Plasmodium falciparum malaria.” Nature communications vol. 6 8170. 8 Sep. 2015, doi:10.1038/ncomms9170 |
| Not burden estimation, describing the relationship between the population prevalence and clinical incidence of vivax malaria | Battle, Katherine E et al. “Defining the relationship between Plasmodium vivax parasite rate and clinical disease.” Malaria journal vol. 14 191. 7 May. 2015, doi:10.1186/s12936-015-0706-3 |
| Generated risk map, not direct burden result | Assaré, Rufin K et al. “The spatial distribution of Schistosoma mansoni infection in four regions of western Côte d'Ivoire.” Geospatial health vol. 10,1 345. 3 Jun. 2015, doi:10.4081/gh.2015.345 |
| Generated risk map, not direct burden result | Pigott, David M et al. “Global distribution maps of the leishmaniases.” eLife vol. 3 e02851. 27 Jun. 2014, doi:10.7554/eLife.02851 |
| Analysis from GBD | Murray, Christopher J L et al. “Global, regional, and national incidence and mortality for HIV, tuberculosis, and malaria during 1990-2013: a systematic analysis for the Global Burden of Disease Study 2013.” Lancet (London, England) vol. 384,9947 (2014): 1005-70. doi:10.1016/S0140-6736(14)60844-8 |
| Generated risk map and estimated population at risk not direct burden result | Caminade, Cyril et al. “Impact of climate change on global malaria distribution.” Proceedings of the National Academy of Sciences of the United States of America vol. 111,9 (2014): 3286-91. doi:10.1073/pnas.1302089111 |
| Predicted prevalence not direct burden result | Woodhall, Dana M et al. “Use of geospatial modeling to predict Schistosoma mansoni prevalence in Nyanza Province, Kenya.” PloS one vol. 8,8 e71635. 14 Aug. 2013, doi:10.1371/journal.pone.0071635 |
| Mapped risk not direct burden result | Raso, Giovanna et al. “Mapping malaria risk among children in Côte d'Ivoire using Bayesian geo-statistical models.” Malaria journal vol. 11 160. 9 May. 2012, doi:10.1186/1475-2875-11-160 |
| Calculating the alignment factor, no burden result | Schur, Nadine et al. “Modelling age-heterogeneous Schistosoma haematobium and S. mansoni survey data via alignment factors.” Parasites & vectors vol. 4 142. 20 Jul. 2011, doi:10.1186/1756-3305-4-142 |
| Predicted prevalence not direct burden result | Haque, Ubydul et al. “Spatial prediction of malaria prevalence in an endemic area of Bangladesh.” Malaria journal vol. 9 120. 9 May. 2010, doi:10.1186/1475-2875-9-120 |
| Exploring the relationship between parasite rate and incidence, not burden estimation | Patil, Anand P et al. “Defining the relationship between Plasmodium falciparum parasite rate and clinical disease: statistical models for disease burden estimation.” Malaria journal vol. 8 186. 5 Aug. 2009, doi:10.1186/1475-2875-8-186 |
| Comparison of different data collection, not burden estimation | Mathieu, Els et al. “Collecting baseline information for national morbidity alleviation programs: different methods to estimate lymphatic filariasis morbidity prevalence.” The American journal of tropical medicine and hygiene vol. 78,1 (2008): 153-8. |
| GBD data analysis | Roca-Feltrer, Arantxa et al. “Estimates of the burden of malaria morbidity in Africa in children under the age of 5 years.” Tropical medicine & international health : TM & IH vol. 13,6 (2008): 771-83. doi:10.1111/j.1365-3156.2008.02076.x |
| Schistosomiasis is not acute febrile disease | van der Werf, Marieke J et al. “Quantification of clinical morbidity associated with schistosome infection in sub-Saharan Africa.” Acta tropica vol. 86,2-3 (2003): 125-39. doi:10.1016/s0001-706x(03)00029-9 |
| Helminthic is not acute febrile disease | Brooker, S et al. “Estimating the number of helminthic infections in the Republic of Cameroon from data on infection prevalence in schoolchildren.” Bulletin of the World Health Organization vol. 78,12 (2000): 1456-65. |
| Not tropical | Angulo, Frederick J et al. “Incidence of symptomatic Lyme borreliosis in nine European countries.” International journal of infectious diseases : IJID : official publication of the International Society for Infectious Diseases vol. 149 (2024): 107242. doi:10.1016/j.ijid.2024.107242 |
| Not acute | Barrett, Carrie et al. “The national distribution of lymphatic filariasis cases in Malawi using patient mapping and geostatistical modelling.” PLoS neglected tropical diseases vol. 18,3 e0012056. 25 Mar. 2024, doi:10.1371/journal.pntd.0012056 |
| Not tropical | Berke, Olaf et al. “Of Lyme disease and machine learning in a One Health world.” American journal of veterinary research vol. 86,S1 S80-S83. 11 Feb. 2025, doi:10.2460/ajvr.24.10.0300 |
| Not tropical | Brestrich, Gordon et al. “Using meta-analysis to estimate the incidence of Lyme borreliosis clinical manifestations in Denmark, Ireland and Sweden based on publicly-available Lyme neuroborreliosis data.” Ticks and tick-borne diseases vol. 16,4 (2025): 102509. doi:10.1016/j.ttbdis.2025.102509 |
| No model for burden, a systematic review | Chen, Lin H et al. “Epidemiology and burden of dengue fever in the United States: a systematic review.” Journal of travel medicine vol. 30,7 (2023): taad127. doi:10.1093/jtm/taad127 |
| Not tropical | Colby, Emily et al. “Estimated incidence of symptomatic Lyme borreliosis cases in five southern coastal counties in Norway, 2022.” APMIS : acta pathologica, microbiologica, et immunologica Scandinavica vol. 132,11 (2024): 832-842. doi:10.1111/apm.13475 |
| Dalys estimate, no further model | de Amorin Vilharba, Bruna Luiza et al. “Disease burden of congenital Zika virus syndrome in Brazil and its association with socioeconomic data.” Scientific reports vol. 13,1 11882. 23 Jul. 2023, doi:10.1038/s41598-023-38553-4 |
| Estimated averted outcomes and does not report or analyse standalone baseline burden estimates, the baseline are calibrated to observed outbreak trajectories | Delport, Dominic et al. “Estimating the historical impact of outbreak response immunisation programmes across 210 outbreaks in low and middle-income countries.” BMJ global health vol. 10,7 e016887. 9 Jul. 2025, doi:10.1136/bmjgh-2024-016887 |
| Not acute disease | Ledien, Julia et al. “From serological surveys to disease burden: a modelling pipeline for Chagas disease.” Philosophical transactions of the Royal Society of London. Series B, Biological sciences vol. 378,1887 (2023): 20220278. doi:10.1098/rstb.2022.0278 |
| Temperature related burden based on reported data | Seposo, Xerxes et al. “Projecting temperature-related dengue burden in the Philippines under various socioeconomic pathway scenarios.” Frontiers in public health vol. 12 1420457. 23 Dec. 2024, doi:10.3389/fpubh.2024.1420457 |
| Economic burden | Shepard, Donald S et al. “Economic evaluation of Wolbachia deployment in Colombia: A modeling study.” PloS one vol. 20,4 e0307045. 30 Apr. 2025, doi:10.1371/journal.pone.0307045 |
| WHO dalys calculation | Yang, Guo-Jing et al. “Discrepancies in neglected tropical diseases burden estimates in China: comparative study of real-world data and Global Burden of Disease 2021 data (2004-2020).” BMJ (Clinical research ed.) vol. 388 e080969. 18 Feb. 2025, doi:10.1136/bmj-2024-080969 |
| Direct data from national system | Zhang, Yun-Fei et al. “Zoonotic diseases in China: epidemiological trends, incidence forecasting, and comparative analysis between real-world surveillance data and Global Burden of Disease 2021 estimates.” Infectious diseases of poverty vol. 14,1 60. 4 Jul. 2025, doi:10.1186/s40249-025-01335-3 |
| GBD data analysis | Zheng, Jinxin et al. “Global burden of malaria and neglected tropical diseases in children and adolescents, 1990-2019: a population-based, cross-sectional study.” Journal of the Royal Society of Medicine vol. 118,3 (2025): 82-96. doi:10.1177/01410768251321572 |
| No model for burden estimation | Deplazes, P., et al. "Global distribution of alveolar and cystic echinococcosis." Advances in parasitology 95 (2017): 315-493. |
| Economic burden | Shepard, Donald S., et al. "The global economic burden of dengue: a systematic analysis." The Lancet infectious diseases 16.8 (2016): 935-941. |
| Systematic review, no model | Noguera Z, Liz P., et al. "The dual burden of animal and human zoonoses: A systematic review." PLoS neglected tropical diseases 16.10 (2022): e0010540. |
| Cluster survey, not burden estimation | Nagavarapu, Sudha, et al. "Disease burden estimation of lymphatic filariasis in an endemic area." Lymphatic Research and Biology 20.3 (2022): 319-324. |
| Reported data, standard dalys calculation | Hung, Trinh Manh, et al. "The estimates of the health and economic burden of dengue in Vietnam." Trends in parasitology 34.10 (2018): 904-918. |
| Standard dalys calculation | Vidal, Emily Raquel Nunes, et al. "Epidemiological burden of Chikungunya fever in Brazil, 2016 and 2017." Tropical Medicine & International Health 27.2 (2022): 174-184. |
| Review, no model | Noguera Zayas, Liz Paola, Simon Rueegg, and Paul Torgerson. "The burden of zoonoses in Paraguay: A systematic review." PLOS Neglected Tropical Diseases 15.11 (2021): e0009909. |
| Review, no model | Bitam, Idir, et al. "Fleas and flea-borne diseases." International journal of infectious diseases 14.8 (2010): e667-e676. |
| No model, economic burden | Mekonnen, Sefinew Alemu, et al. "Health and economic burden of foodborne zoonotic diseases in Amhara region, Ethiopia." PLoS One 16.12 (2021): e0262032. |
| Standard dalys calculation, economic burden | Luh, Dih-Ling, et al. "Economic cost and burden of dengue during epidemics and non-epidemic years in Taiwan." Journal of infection and public health 11.2 (2018): 215-223. |
| GBD guideline \| no model | Puntasecca, Christopher J., Charles H. King, and Angelle Desiree LaBeaud. "Measuring the global burden of chikungunya and Zika viruses: A systematic review." PLoS neglected tropical diseases 15.3 (2021): e0009055. |
| Not communicable disease | Frings, Michael, et al. "Modeling and mapping the burden of disease in Kenya." Scientific reports 8.1 (2018): 9826. |
| Review, no model | Counotte, Michel J., et al. "The burden of zoonoses in Kyrgyzstan: a systematic review." PLoS neglected tropical diseases 10.7 (2016): e0004831. |
| GBD dalys | Wachira, Virginia Kagure, et al. "Burden of Disease of Guillain–Barré Syndrome in Brazil before and during the Zika virus epidemic 2014–2016." Tropical Medicine & International Health 26.1 (2021): 66-81. |
| Review | LI, Yi-Ting, et al. "Global burden and challenges of parasitic diseases in Africa." Chinese Journal of Schistosomiasis Control 30.2 (2018): 226. |
| Review, no model | Christou, Leonidas. "The global burden of bacterial and viral zoonotic infections." Clinical Microbiology and Infection 17.3 (2011): 326-330. |
| Standard dalys calculation, economic burden | Sultanov, Akmetzhan A., et al. "Rabies in Kazakhstan." PLoS neglected tropical diseases 10.8 (2016): e0004889. |
| Review, standard dalys calculation | Jordan, H., et al. "Costing the Morbidity and Mortality Consequences of Zoonoses Using Health‐Adjusted Life Years." Transboundary and emerging diseases 63.5 (2016): e301-e312. |
| Approach review | Shepard, Donald S., et al. "Approaches to refining estimates of global burden and economics of dengue." PLoS neglected tropical diseases 8.11 (2014): e3306. |
| Review, no model | Conners, Erin E., et al. "A global systematic review of Chagas disease prevalence among migrants." Acta tropica 156 (2016): 68-78. |
| Population attributable risk measurement, standard dalys calculation | Sanhueza, Juan M., et al. "Estimation of the burden of leptospirosis in New Zealand." Zoonoses and public health 67.2 (2020): 167-176. |
| Standard dalys calculation, economic burden | Singh, B. B., et al. "Estimation of the health and economic burden of neurocysticercosis in India." Acta tropica 165 (2017): 161-169. |
| Standard dalys calculation | Nujum, Zinia T., et al. "Burden of dengue in Kerala using disability-adjusted life years from 2006 to 2016." Indian Journal of Public Health 63.2 (2019): 107-113. |
| Review | Lalloo, David G., Peju Olukoya, and Piero Olliaro. "Malaria in adolescence: burden of disease, consequences, and opportunities for intervention." The Lancet infectious diseases 6.12 (2006): 780-793. |
| Review | Kalil-Filho, Roberto. "Globalization of Chagas disease burden and new treatment perspectives." Journal of the American College of Cardiology 66.10 (2015): 1190-1192. |
| Review \| wrong study design | Ohta, Nobuo, and Jitra Waikagul. "Disease burden and epidemiology of soil-transmitted helminthiases and schistosomiasis in Asia: the Japanese perspective." Trends in parasitology 23.1 (2007): 30-35.. |
| Mathematical model review | Atkinson, Jo-An M., et al. "Synthesising 30 years of mathematical modelling of Echinococcus transmission." PLoS neglected tropical diseases 7.8 (2013): e2386. |
| Life quality, not direct health burden | Schulte, Annika, et al. "Health-related quality of life after dengue fever, Morelos, Mexico, 2016–2017." Emerging infectious diseases 26.4 (2020): 751. |
| Standard dalys calculation and economic burden | Undurraga, Eduardo A., et al. "Economic and disease burden of dengue in Mexico." PLoS neglected tropical diseases 9.3 (2015): e0003547. |
| Review \| wrong outcome | Kale, O. O. "Onchocerciasis: the burden of disease." Annals of Tropical Medicine & Parasitology 92.sup1 (1998): S101-S115. |
| Official national surveillance reporting data, no model applied | Torres, Jaime R., et al. "Epidemiology and disease burden of pediatric dengue in Venezuela." Journal of the Pediatric Infectious Diseases Society 4.4 (2015): 288-289.. |
| Standard dalys calculation | Mora-Salamanca, Andrés Felipe, Alexandra Porras-Ramírez, and Fernando Pío de la Hoz Restrepo. "Burden of disease due to microcephaly associated with the Zika virus in Colombia." Cadernos de saude publica 36 (2020): e00215319. |
| Standard dalys calculation | Šmit, Renata, and Maarten J. Postma. "The burden of tick-borne encephalitis in disability-adjusted life years (DALYs) for Slovenia." PLoS One 10.12 (2015): e0144988. |
| Standard dalys calculation | Robb, John, et al. "The greatest health problem of the Middle Ages? Estimating the burden of disease in medieval England." International Journal of Paleopathology 34 (2021): 101-112. |
| Review | Lewison, Grant, and Divya Srivastava. "Malaria research, 1980–2004, and the burden of disease." Acta Tropica 106.2 (2008): 96-103. |
| Review | Fazaludeen Koya, Shaffi, et al. "Vector-borne and zoonotic diseases in the Eastern Mediterranean Region: a systematic review." Journal of Epidemiology and Global Health 13.1 (2023): 105-114. |
| Review | Williams, Thomas N., and David J. Weatherall. "World distribution, population genetics, and health burden of the hemoglobinopathies." Cold Spring Harbor perspectives in medicine 2.9 (2012): a011692. |
| GBD result analysis | Heydarpour, Fatemeh, et al. "Incidence and disability-adjusted life years (Dalys) attributable to leishmaniasis in Iran, 2013." Ethiopian journal of health sciences 26.4 (2016): 381-388. |
| Review | Karshima, Solomon Ngutor, et al. "Africa-wide meta-analysis on the prevalence and distribution of human cystic echinococcosis and canine Echinococcus granulosus infections." Parasites & Vectors 15.1 (2022): 357. |
| Standard dalys calculation | van den Wijngaard, Cees C., et al. "The burden of Lyme borreliosis expressed in disability-adjusted life years." The European Journal of Public Health 25.6 (2015): 1071-1078. |
| Extracted from GBD | Scheufele, Christian J., Rachel L. Giesey, and Gregory R. Delost. "The global, regional, and national burden of leishmaniasis: An ecologic analysis from the Global Burden of Disease Study 1990-2017." Journal of the American Academy of Dermatology 84.4 (2021): 1203-1205. |
| Reported case data and used WHO DALY calculation method | Cardona-Ospina, Jaime A., et al. "Estimating the burden of disease and the economic cost attributable to chikungunya, Colombia, 2014." Transactions of the Royal Society of Tropical Medicine and Hygiene 109.12 (2015): 793-802. |
| No model, descriptive result | Halsby, K. D., et al. "The health burden of orphan zoonotic disease in the United Kingdom, 2005–2009." Zoonoses and public health 61.1 (2014): 39-47. |
| Extracted from GBD | Wang, Yuxin, et al. "Burden of malaria in Sao Tome and Principe, 1990–2019: findings from the global burden of disease study 2019." International journal of environmental research and public health 19.22 (2022): 14817. |
| Reported case data and used WHO DALY calculation method | Beauté, Julien, and Sirenda Vong. "Cost and disease burden of dengue in Cambodia." BMC public health 10.1 (2010): 521. |
| Review | Cibulskis, Richard E., et al. "Estimating trends in the burden of malaria at country level." Defining and Defeating the Intolerable Burden of Malaria III: Progress and Perspectives: Supplement to Volume 77 (6) of American Journal of Tropical Medicine and Hygiene (2007). |
| Disability weight, not health burden result | Jia, Tie-Wu, et al. "Quantifying quality of life and disability of patients with advanced schistosomiasis japonica." PLoS neglected tropical diseases 5.2 (2011): e966. |
| Reported case data and used standard DALY calculation method | Luz, P. M., B. Grinsztejn, and A. P. Galvani. "Disability adjusted life years lost to dengue in Brazil." Tropical Medicine & International Health 14.2 (2009): 237-246. |
| Analysis using data extracted from GBD | Stanaway, Jeffrey D., et al. "The global burden of dengue: an analysis from the Global Burden of Disease Study 2013." The Lancet infectious diseases 16.6 (2016): 712-723. |
| Economic burden, and standard DALY calculation | Tiga, D. Carolina, et al. "Persistent symptoms of dengue: estimates of the incremental disease and economic burden in Mexico." The American journal of tropical medicine and hygiene 94.5 (2016): 1085. |
| Review | Finkelstein, Julia L., et al. "Decision-model estimation of the age-specific disability weight for schistosomiasis japonica: a systematic review of the literature." PLoS neglected tropical diseases 2.3 (2008): e158. |
| Reported burden, no model | Colpani, Agnese, et al. "Trends in the Surgical Incidence of Cystic Echinococcosis in Uzbekistan from 2011 to 2018." The American Journal of Tropical Medicine and Hygiene 106.2 (2021): 724. |
| Zinc deficiency, not tropical acute infectious disease | Fischer Walker, C. L., M. Ezzati, and R. E. Black. "Global and regional child mortality and burden of disease attributable to zinc deficiency." European journal of clinical nutrition 63.5 (2009): 591-597. |
| No model, standard DALY calculation | Grace, Delia, et al. "The multiple burdens of zoonotic disease and an ecohealth approach to their assessment." Tropical animal health and production 44.Suppl 1 (2012): 67-73. |
| Social burden | Jones, Caroline OH, and Holly A. Williams. "The social burden of malaria: what are we measuring?." The American journal of tropical medicine and hygiene 71.2 Supp (2004): 156-161. |
| Review, not health burden result | Wang, Xin, et al. "Using a One Health approach to prioritize zoonotic diseases in China, 2019." PLoS One 16.11 (2021): e0259706. |
| No model, review | Rodriguez-Morales, Alfonso J., Wilmer E. Villamil-Gómez, and Carlos Franco-Paredes. "The arboviral burden of disease caused by co-circulation and co-infection of dengue, chikungunya and Zika in the Americas." Travel medicine and infectious disease 14.3 (2016): 177-179. |
| Extracted from GBD | Martins-Melo, Francisco Rogerlândio, et al. "Burden of Chagas disease in Brazil, 1990–2016: findings from the Global Burden of Disease Study 2016." International journal for parasitology 49.3-4 (2019): 301-310. |
| Reported burden, standard dalys calculation | Abdalla, Safa I., Elfatih M. Malik, and Kamil M. Ali. "The burden of malaria in Sudan: incidence, mortality and disability–adjusted life–years." Malaria journal 6.1 (2007): 97. |
| Extracted from GBD | Mohan, Sakshi, et al. "Estimating the global demand curve for a leishmaniasis vaccine: A generalisable approach based on global burden of disease estimates." PLoS Neglected Tropical Diseases 16.6 (2022): e0010471. |
| Reported burden, standard dalys calculation | Seyler, T., et al. "Estimating the burden of disease and the economic cost attributable to chikungunya, Andhra Pradesh, India, 2005–2006." Transactions of the Royal Society of Tropical Medicine and Hygiene 104.2 (2010): 133-138. |
| Not communicable disease | Murray, R., et al. "Estimates of the burden of illness for eight enteric pathogens associated with animal contact in Canada." Epidemiology & Infection 145.16 (2017): 3413-3423. |
| Reported burden, standard dalys calculation | Wettstein, Zachary S., et al. "Total economic cost and burden of dengue in Nicaragua: 1996–2010." The American journal of tropical medicine and hygiene 87.4 (2012): 616. |
| Using the Burden of Communicable Diseases in Europe approach | Fafangel, Mario, et al. "Estimating the annual burden of tick-borne encephalitis to inform vaccination policy, Slovenia, 2009 to 2013." Eurosurveillance 22.16 (2017): 30509. |
| Reported burden, standard dalys calculation | Gunda, Resign, Moses John Chimbari, and Samson Mukaratirwa. "Assessment of burden of malaria in Gwanda District, Zimbabwe, using the disability adjusted life years." International Journal of Environmental Research and Public Health 13.2 (2016): 244. |
| Review | Cox, Jonathan, et al. "The uncertain burden of Plasmodium falciparum epidemics in Africa." TRENDS in Parasitology 23.4 (2007): 142-148. |
| Review | Garcia, Melissa N., et al. "Historical perspectives on the epidemiology of human Chagas disease in Texas and recommendations for enhanced understanding of clinical Chagas disease in the southern United States." PLoS neglected tropical diseases 9.11 (2015): e0003981. |
| Reported burden, standard dalys calculation | Krishnamoorthy, K., et al. "Burden of chikungunya in India: estimates of disability adjusted life years (DALY) lost in 2006 epidemic." Journal of vector borne diseases 46.1 (2009): 26. |
| Finding from GBD | Badirzadeh, Alireza, et al. "Burden of malaria in Iran, 1990–2010: findings from the Global Burden of Disease Study 2010." Archives of Iranian Medicine 19.4 (2016): 241-247. |
| Review | Gilmour, Beth, et al. "The prevalence of tuberculosis, malaria and soil-transmitted helminth infection in minority indigenous people of Southeast Asia and the Western Pacific: protocol for a systematic review and meta-analysis." Systematic reviews 10.1 (2021): 203. |
| Reported burden, standard dalys calculation | Yang, Li-Ping, et al. "Burden of disease measured by disability-adjusted life years and a disease forecasting time series model of scrub typhus in Laiwu, China." PLoS neglected tropical diseases 9.1 (2015): e3420. |
| Standard dalys calculation | Praet, Nicolas, et al. "The disease burden of Taenia solium cysticercosis in Cameroon." PLoS neglected tropical diseases 3.3 (2009): e406. |
| Extracted from GBD | Montresor, Antonio, et al. "Reduction in DALYs lost due to soil-transmitted helminthiases and schistosomiasis from 2000 to 2019 is parallel to the increase in coverage of the global control programmes." PLoS neglected tropical diseases 16.7 (2022): e0010575. |
| Extracted from GBD | Bezerra, Juliana Maria Trindade, et al. "Burden of leishmaniasis in Brazil and federated units, 1990-2016: Findings from Global Burden of Disease Study 2016." PLoS neglected tropical diseases 12.9 (2018): e0006697. |
| Wrong outcome, economic burden | Rafique, Ibrar, et al. "Economic burden of dengue in four major cities of Pakistan during 2011." Hospital 6.5 (2015): 3-15. |
| No model for burden estimation | Albajar-Vinas, P., and J. Jannin. "The hidden Chagas disease burden in Europe." Eurosurveillance 16.38 (2011). |
| Economic burden | GAO, Yue-Xia, et al. "Disease burden of patients with advanced schistosomiasis in Jiangsu Province." Chinese Journal of Schistosomiasis Control 30.5 (2018): 552. |
| Reported burden, no model | Facciolà, Alessio, et al. "Brucellosis is a public health problem in southern Italy: Burden and epidemiological trend of human and animal disease." Journal of infection and public health 11.6 (2018): 861-866. |
| GBD dalys calculation method | LI, Yi-Ting, et al. "Approaches used for assessment of the burden of advanced schistosomiasis japonica: a comparative study." Chinese Journal of Schistosomiasis Control 31.3 (2019): 280. |
| Calculated expansion factors, no model for burden estimation, | Vong, S., et al. "Under-recognition and reporting of dengue in Cambodia: a capture–recapture analysis of the National Dengue Surveillance System." Epidemiology & Infection 140.3 (2012): 491-499. |
| Extracted from GBD | Bezerra, Juliana Maria Trindade, et al. "Changes in malaria patterns in Brazil over 28 years (1990–2017): results from the Global Burden of Disease Study 2017." Population Health Metrics 18.Suppl 1 (2020): 5. |
| No model, extracted from GBD | Deribew, Amare, et al. "Incidence, prevalence and mortality rates of malaria in Ethiopia from 1990 to 2015: analysis of the global burden of diseases 2015." Malaria journal 16.1 (2017): 271. |
| Review | Hotez, Peter J., et al. "The neglected tropical diseases of Latin America and the Caribbean: a review of disease burden and distribution and a roadmap for control and elimination." PLoS neglected tropical diseases 2.9 (2008): e300. |
| Not in English, extracted from GBD | Araújo, Valdelaine Etelvina Miranda de, et al. "Increase in the burden of dengue in Brazil and federated units, 2000 and 2015: analysis of the Global Burden of Disease Study 2015." Revista brasileira de epidemiologia 20 (2017): 205-216. |
| Standard dalys calculation method | Anderson, Katie B., et al. "Burden of symptomatic dengue infection in children at primary school in Thailand: a prospective study." The Lancet 369.9571 (2007): 1452-1459. |
| Standard dalys calculation method | Fèvre, Eric M., et al. "Estimating the burden of rhodesiense sleeping sickness during an outbreak in Serere, eastern Uganda." BMC Public Health 8.1 (2008): 96. |
| Standard dalys calculation method | Jia, Tie-Wu, et al. "Burden of disease in schistosomiasis japonica I Calculation and evaluation of years lived with disability of chronic schistosomiasis." Chinese Journal of Schistosomiasis Control 23.3 (2011): 243. |
| National reported data analysis | Olveda, Remigio M., et al. "National survey data for zoonotic schistosomiasis in the Philippines grossly underestimates the true burden of disease within endemic zones: implications for future control." International Journal of Infectious Diseases 45 (2016): 13-17. |
| A survey, no model | Cho Min, Naing. "Assessment of dengue hemorrhagic fever in Myanmar." Southeast Asian J Trop Med Public Health 31.4 (2000): 636-41. |
| Extracted from GBD | Wang, Haidong, et al. "Global, regional, national, and selected subnational levels of stillbirths, neonatal, infant, and under-5 mortality, 1980–2015: a systematic analysis for the Global Burden of Disease Study 2015." The Lancet 388.10053 (2016): 1725-1774. |
| No model, Standard dalys calculation method | Cardona-Ospina, Jaime Andrés, Fredi Alexander Diaz-Quijano, and Alfonso J. Rodríguez-Morales. "Burden of chikungunya in Latin American countries: estimates of disability-adjusted life-years (DALY) lost in the 2014 epidemic." International journal of infectious diseases 38 (2015): 60-61. |
| Review, no model | Bailey, Freddie, et al. "A new perspective on cutaneous leishmaniasis—Implications for global prevalence and burden of disease estimates." PLoS neglected tropical diseases 11.8 (2017): e0005739. |
| No model, Standard dalys calculation method | Cardona-Ospina, Jaime A., Alfonso J. Rodriguez-Morales, and Wilmer E. Villamil-Gómez. "The burden of Chikungunya in one coastal department of Colombia (Sucre): Estimates of the disability adjusted life years (DALY) lost in the 2014 epidemic." Journal of infection and public health 8.6 (2015): 644-646. |
| Reported disease burden, economic burden | Wang, X. X., Y. X. Li, and Z. D. Yin. "Analysis on disease burdern of Japanese encephalitis (JE) in Gansu province." Zhongguo yi Miao he Mian yi 16.3 (2010): 246-250. |
| No model, using calculated prevalence to get case number | Bern, Caryn, and Susan P. Montgomery. "An estimate of the burden of Chagas disease in the United States." Clinical Infectious Diseases 49.5 (2009): e52-e54. |
| Review, no model | Guinovart, C., et al. "Malaria: burden of disease." Current molecular medicine 6.2 (2006): 137-140. |
| Reported burden, standard dalys calculation | Thaw, Ei Ei. "Using disability-adjusted life years to assess the economic impact of dengue in Puerto Rico: 1984-1994." The American journal of tropical medicine and hygiene (1998). |
| A previously developed model and standard dalys calculation | Mathew, Christopher G., et al. "The health and economic burdens of lymphatic filariasis prior to mass drug administration programs." Clinical Infectious Diseases 70.12 (2020): 2561-2567. |
| Standard dalys calculation | Rodríguez, Raúl Castro, et al. "The burden of dengue and the financial cost to Colombia, 2010–2012." The American journal of tropical medicine and hygiene 94.5 (2016): 1065. |
| Economic burden, review | Andrade, Mônica V., et al. "The economic burden of malaria: a systematic review." Malaria journal 21.1 (2022): 283. |
| No model applied | Kaur, Jaspreet, et al. "Surveillance-based estimation of the malaria disease burden in a low endemic state of Punjab, India, targeted for malaria elimination." Transactions of The Royal Society of Tropical Medicine and Hygiene 115.5 (2021): 512-519. |
| Systematic review | Geebelen, Laurence, et al. "Combining primary care surveillance and a meta-analysis to estimate the incidence of the clinical manifestations of Lyme borreliosis in Belgium, 2015–2017." Ticks and tick-borne diseases 10.3 (2019): 598-605. |
| Review | Utzinger, Jürg, and Jennifer Keiser. "Schistosomiasis and soil-transmitted helminthiasis: common drugs for treatment and control." Expert opinion on pharmacotherapy 5.2 (2004): 263-285. |
| Extracted from GBD | Mendoza-Cano, Oliver, et al. "The burden of dengue in children by calculating spatial temperature: a methodological approach using remote sensing techniques." International Journal of Environmental Research and Public Health 18.8 (2021): 4230. |
| Standard dalys calculation | Vásquez-Trujillo, Adolfo, et al. "Burden of dengue in the State of Meta, Colombia (2010-2016)." Cadernos de Saúde Pública 36 (2020): e00055119. |
| Review | Fenwick, A. "The global burden of neglected tropical diseases." Public health 126.3 (2012): 233-236. |
| Descriptive review | Wartel, T. Anh, et al. "Three decades of dengue surveillance in five highly endemic South East Asian countries: a descriptive review." Asia Pacific Journal of Public Health 29.1 (2017): 7-16. |
| Standard dalys calculation | Feldstein, Leora R., et al. "Estimating the cost of illness and burden of disease associated with the 2014–2015 chikungunya outbreak in the US Virgin Islands." PLoS neglected tropical diseases 13.7 (2019): e0007563. |
| No model applied, sampling to adjust fatality rate | Renschler, John P., et al. "Estimated under-five deaths associated with poor-quality antimalarials in sub-Saharan Africa." The American journal of tropical medicine and hygiene 92.Suppl 6 (2015): 119. |
| Wrong outcome (Force of Infection), not direct health burden | Imai, Natsuko, et al. "Estimating dengue transmission intensity from sero-prevalence surveys in multiple countries." PLoS neglected tropical diseases 9.4 (2015): e0003719. |
| Reported burden and estimated the under-reporting rate | Reid, H., et al. "Assessment of the burden of human African trypanosomiasis by rapid participatory appraisal in three high-risk villages in Urambo District, Northwest Tanzania." (2012). |
| Reported burden | Iyer, Veena, et al. "Infectious disease burden in Gujarat (2005–2011): comparison of selected infectious disease rates with India." Emerging health threats journal 7.1 (2014): 22838. |
| Wrong outcome (probability of infection outcome), not direct health burden | Johansson, Michael A., Pedro FC Vasconcelos, and J. Erin Staples. "The whole iceberg: estimating the incidence of yellow fever virus infection from the number of severe cases." Transactions of the Royal Society of Tropical Medicine and Hygiene 108.8 (2014): 482-487. |
| Not direct health burden | Mwanziva, Charles, et al. "Defining malaria burden from morbidity and mortality records, self treatment practices and serological data in Magugu, Babati District, northern Tanzania." Tanzania journal of health research 13.2 (2011): 93-96. |
| Mathematical model \| measure intervention | Turner, Hugo C., et al. "Modelling the impact of ivermectin on River Blindness and its burden of morbidity and mortality in African Savannah: EpiOncho projections." Parasites & Vectors 7.1 (2014): 241. |
| Review | Devleesschauwer, Brecht, et al. "The burden of parasitic zoonoses in Nepal: a systematic review." PLoS neglected tropical diseases 8.1 (2014): e2634. |
| Mathematical model \| measure intervention | Penny, Melissa A., et al. "Public health impact and cost-effectiveness of the RTS, S/AS01 malaria vaccine: a systematic comparison of predictions from four mathematical models." The Lancet 387.10016 (2016): 367-375. |
| Review | Samba, E. M. "The burden of malaria in Africa." (1997): 17. |
| Review | Kamau, Alice, et al. "A systematic review of changing malaria disease burden in sub-Saharan Africa since 2000: comparing model predictions and empirical observations." BMC medicine 18.1 (2020): 94. |
| Review | Sykes, Robert A., and Phoebe Makiello. "An estimate of Lyme borreliosis incidence in Western Europe." Journal of public health 39.1 (2017): 74-81. |
| Not direct health burden (malaria transmission intensity) | Stewart, Laveta, et al. "Rapid assessment of malaria transmission using age-specific sero-conversion rates." PloS one 4.6 (2009): e6083. |
| Review | Attaway, David Frost, et al. "Assessing the methods needed for improved dengue mapping: a SWOT analysis." The Pan African Medical Journal 17 (2014): 289. |
| Treatment measurement, standard dalys calculation method | Fernandes, Silke, et al. "Cost-effectiveness of two versus three or more doses of intermittent preventive treatment for malaria during pregnancy in sub-Saharan Africa: a modelling study of meta-analysis and cost data." The Lancet Global Health 3.3 (2015): e143-e153. |
| Passive surveillance and survey, no model applied | Lim, Jacqueline Kyungah, et al. "Evaluating dengue burden in Africa in passive fever surveillance and seroprevalence studies: protocol of field studies of the Dengue Vaccine Initiative." BMJ open 8.1 (2018): e017673. |
| Not direct health burden, pregnancies at risk | Dellicour, Stephanie, et al. "Quantifying the number of pregnancies at risk of malaria in 2007: a demographic study." PLoS medicine 7.1 (2010): e1000221. |
| Cross-sectional survey, not burden estimation model | Chepkorir, Edith, et al. "Serological evidence of Flavivirus circulation in human populations in Northern Kenya: an assessment of disease risk 2016–2017." Virology journal 16.1 (2019): 65. |
| Review | Cucunuba, Zulma M., et al. "Increased mortality attributed to Chagas disease: a systematic review and meta-analysis." Parasites & vectors 9.1 (2016): 42. |
| Wrong outcome (determine associations between outcome and variables) | Chitunhu, Simangaliso, and Eustasius Musenge. "Spatial and socio-economic effects on malaria morbidity in children under 5 years in Malawi in 2012." Spatial and Spatio-temporal Epidemiology 16 (2016): 21-33. |
| Review | Oh, Jin-Kyoung, and Elisabete Weiderpass. "Infection and cancer: global distribution and burden of diseases." Annals of global health 80.5 (2014): 384-392. |
| Review \| economic burden | Castro, Marcia C., Mary E. Wilson, and David E. Bloom. "Disease and economic burdens of dengue." The Lancet Infectious Diseases 17.3 (2017): e70-e78. |
| Not tropical acute infectious disease | Parkin, Donald Maxwell. "The global health burden of infection‐associated cancers in the year 2002." International journal of cancer 118.12 (2006): 3030-3044. |
| Estimating female worm burdens, not human burden | Neves, M. Inês, Joanne P. Webster, and Martin Walker. "Estimating helminth burdens using sibship reconstruction." Parasites & Vectors 12.1 (2019): 441. |
| Narrative literature review | Franklinos, Lydia HV, et al. "The effect of global change on mosquito-borne disease." The Lancet infectious diseases 19.9 (2019): e302-e312. |
| Standard dalys calculation method | Prüss-Ustün, Annette, et al. "Burden of disease from inadequate water, sanitation and hygiene for selected adverse health outcomes: An updated analysis with a focus on low-and middle-income countries." International journal of hygiene and environmental health 222.5 (2019): 765-777. |
| Reported case data | Slunge, Daniel, Anders Boman, and Marie Studahl. "Burden of tick-borne encephalitis, Sweden." Emerging Infectious Diseases 28.2 (2022): 314. |
| Review | Tsuji, Naotoshi. "Schistosomiasis and hookworm infection in humans: Disease burden, pathobiology and anthelmintic vaccines." Parasitology international 75 (2020): 102051. |
| Standard dalys calculation method | Šmit, Renata. "Reviewing estimates of the burden in disability-adjusted life years (DALYs) of tick-borne encephalitis in Slovenia." Expert review of pharmacoeconomics & outcomes research 19.3 (2019): 299-303. |
| Review | Li, Qin, and Xiao ZHOU. "Progress of researches on approaches for estimating the burden of vector⁃ borne diseases." Chinese Journal of Schistosomiasis Control 34.1 (2022): 95. |
| Describe the epidemiology, no model | Halsby, K., et al. "Epidemiology of toxocariasis in England and Wales." Zoonoses and public health 63.7 (2016): 529-533. |
| Personal View paper | Mordecai, Erin A., et al. "Climate change could shift disease burden from malaria to arboviruses in Africa." The Lancet Planetary Health 4.9 (2020): e416-e423. |
| Review | Battle, Katherine E., et al. "The global public health significance of Plasmodium vivax." Advances in parasitology 80 (2012): 1-111. |
| Cross-sectional study, no model | Wang, Ran, et al. "The epidemiology and disease burden of children hospitalized for viral infections within the family Flaviviridae in China: A national cross-sectional study." PLoS neglected tropical diseases 16.7 (2022): e0010562. |
| Review | Bern, Caryn, James H. Maguire, and Jorge Alvar. "Complexities of assessing the disease burden attributable to leishmaniasis." PLoS neglected tropical diseases 2.10 (2008): e313. |
| Review | Murthy, J. M. K., et al. "Rabies, tetanus, leprosy, and malaria." Handbook of Clinical Neurology 121 (2014): 1501-1520. |
| Determine association, not burden estimation | Kerridge, Bradley T., et al. "Conflict and diarrheal and related diseases: A global analysis." Journal of epidemiology and global health 3.4 (2013): 269-277. |
| Extracted from GBD | Gómez-Ochoa, Sergio Alejandro, et al. "Global, regional, and national trends of Chagas disease from 1990 to 2019: comprehensive analysis of the global burden of disease study." Global heart 17.1 (2022): 59. |
| Database, not burden estimation | Battle, Katherine E., et al. "Global database of matched Plasmodium falciparum and P. vivax incidence and prevalence records from 1985–2013." Scientific Data 2.1 (2015): 1-12. |
| Risk factor, not burden estimation | Galipó, Erika, et al. "Spatial distribution and risk factors for human cysticercosis in Colombia." Parasites & vectors 14.1 (2021): 590. |
| Extracted from GBD | Li, Zhuo, et al. "Temporal trends in the burden of non-communicable diseases in countries with the highest malaria burden, 1990–2019: Evaluating the double burden of non-communicable and communicable diseases in epidemiological transition." Globalization and Health 18.1 (2022): 90. |
| Extracted from GBD | Bell, David, and Kristian Schultz Hansen. "Relative burdens of the COVID-19, malaria, tuberculosis, and HIV/AIDS epidemics in sub-Saharan Africa." The American journal of tropical medicine and hygiene 105.6 (2021): 1510. |
| WHO methodology | Cronin, Aidan A., et al. "Quantifying the burden of disease associated with inadequate provision of water and sanitation in selected sub-Saharan refugee camps." Journal of water and health 7.4 (2009): 557-568. |
| Standard dalys calculation method, economic burden | Carrasco, Luis R., et al. "Economic impact of dengue illness and the cost-effectiveness of future vaccination programs in Singapore." PLoS neglected tropical diseases 5.12 (2011): e1426. |
| Standard dalys calculation method | Hackett, Finola, et al. "Incorporating scale dependence in disease burden estimates: the case of human African trypanosomiasis in Uganda." PLoS neglected tropical diseases 8.2 (2014): e2704. |
| Review | Torgerson, Paul R., et al. "Epidemiology of Taenia saginata taeniosis/cysticercosis: a systematic review of the distribution in central and western Asia and the Caucasus." Parasites & vectors 12.1 (2019): 175. |
| Review | Bailey, Freddie, et al. "Cutaneous leishmaniasis and co-morbid major depressive disorder: a systematic review with burden estimates." PLoS neglected tropical diseases 13.2 (2019): e0007092. |
| Review | Xu, Zhiwei, et al. "Projecting the future of dengue under climate change scenarios: Progress, uncertainties and research needs." PLoS neglected tropical diseases 14.3 (2020): e0008118. |
| Analysis using data from electronic health information systems | Rodriguez-Fernandez, Rodrigo, et al. "The double burden of disease among mining workers in Papua, Indonesia: at the crossroads between Old and New health paradigms." BMC public health 16.1 (2016): 951. |
| Standard dalys calculation method, economic burden | Godói, Isabella Piassi, et al. "Economic and epidemiological impact of dengue illness over 16 years from a public health system perspective in Brazil to inform future health policies including the adoption of a dengue vaccine." Expert review of vaccines 17.12 (2018): 1123-1133. |
| Review | Lyamuya, Eligius, et al. "A socio-economic approach to one health policy research in Southern Africa." (2012). |
| Analysis using data from national surveillance | Khampapongpane, Bouaphanh, et al. "National dengue surveillance in the Lao People's Democratic Republic, 2006–2012: epidemiological and laboratory findings." Western Pacific surveillance and response journal: WPSAR 5.1 (2014): 7. |
| Economic burden | Clark, Danielle V., et al. "Economic impact of dengue fever/dengue hemorrhagic fever in Thailand at the family and population levels." The American journal of tropical medicine and hygiene 72.6 (2005): 786-791. |
| Measure intervention, not direct burden | Butler, William P., and Donald R. Roberts. "Malaria in the Americas: a model of reemergence." Military medicine 165.12 (2000): 897-902. |
| Standard dalys calculation method, economic burden | Castillo-Riquelme, Marianela, et al. "Modelling geographic variation in the cost-effectiveness of control policies for infectious vector diseases: The example of Chagas disease." Journal of health economics 27.2 (2008): 405-426. |
| Active surveillance, no model applied | Yin, Zundong, et al. "Japanese encephalitis disease burden and clinical features of Japanese encephalitis in four cities in the People's Republic of China." The American journal of tropical medicine and hygiene 83.4 (2010): 766. |
| Review \| no model | Li, Yue-Sheng, et al. "Applications and outcomes of periodic epidemiological surveys for schistosomiasis and related economic evaluation in the People's Republic of China." Acta tropica 96.2-3 (2005): 266-275. |
| Review | Adenowo, Abiola Fatimah, et al. "Impact of human schistosomiasis in sub-Saharan Africa." The Brazilian Journal of Infectious Diseases 19.2 (2015): 196-205. |
| Detect cluster and risk factor, not burden result | Haque, Ubydul, et al. "Spatial malaria epidemiology in Bangladeshi highlands." Malaria journal 8.1 (2009): 185. |
| Review | Cuéllar-Sáenz, Jerson Andrés, et al. "Rickettsioses in Colombia during the 20th century: A historical review." Ticks and Tick-borne Diseases 14.2 (2023): 102118. |
| For conducting detection and attribution analyses, not burden estimation | Ebi, Kristie L., et al. "Detecting and attributing health burdens to climate change." Environmental health perspectives 125.8 (2017): 085004. |
| Annual percent change \| surveillance study | Ding, Cheng, et al. "Malaria in China: a longitudinal population-based surveillance study." Epidemiology & Infection 148 (2020): e37. |
| Prospective cohort study, no model applied | Weigle, Kristen A., et al. "Epidemiology of cutaneous leishmaniasis in Colombia: a longitudinal study of the natural history, prevalence, and incidence of infection and clinical manifestations." Journal of Infectious Diseases 168.3 (1993): 699-708. |
| Prospective cohort study, no model applied | Kari, Komang, et al. "A hospital-based surveillance for Japanese encephalitis in Bali, Indonesia." BMC medicine 4.1 (2006): 8. |
| Cross-sectional surveys, no model applied | Mmbando, Bruno P., et al. "A progressive declining in the burden of malaria in north-eastern Tanzania." Malaria journal 9.1 (2010): 216. |
| Measure intervention | Selemani, Majige, et al. "Assessing the effects of mosquito nets on malaria mortality using a space time model: a case study of Rufiji and Ifakara Health and Demographic Surveillance System sites in rural Tanzania." Malaria journal 15.1 (2016): 257. |
| Prospective surveillance, no model applied | Kamau, Alice, et al. "Malaria infection, disease and mortality among children and adults on the coast of Kenya." Malaria journal 19.1 (2020): 210. |
| Cross-sectional study, no model applied | Mutengo, Mable M., et al. "High Schistosoma mansoni disease burden in a rural district of western Zambia." The American Journal of Tropical Medicine and Hygiene 91.5 (2014): 965. |
| Review | Ganeshkumar, Parasuraman, et al. "Dengue infection in India: A systematic review and meta-analysis." PLoS neglected tropical diseases 12.7 (2018): e0006618. |
| Surveillance, no model applied | Sarti, Elsa, et al. "A comparative study on active and passive epidemiological surveillance for dengue in five countries of Latin America." International Journal of Infectious Diseases 44 (2016): 44-49. |
| Socio-economic burden | Bukachi, Salome A., Simiyu Wandibba, and Isaac K. Nyamongo. "The socio-economic burden of human African trypanosomiasis and the coping strategies of households in the South Western Kenya foci." PLoS neglected tropical diseases 11.10 (2017): e0006002. |
| Measure intervention | Hellewell, Joel, et al. "Using ante-natal clinic prevalence data to monitor temporal changes in malaria incidence in a humanitarian setting in the Democratic Republic of Congo." Malaria journal 17.1 (2018): 312. |
| Cross-sectional survey, no model applied | Karim, Mohammad J., et al. "Developing the first national database and map of lymphatic filariasis clinical cases in Bangladesh: Another step closer to the elimination goals." PLoS Neglected Tropical Diseases 13.7 (2019): e0007542. |
| Not in English | Castillo-Salgado, Carlos. "Geo-epidemiologic mapping in the new public health surveillance. The malaria case in Chiapas, Mexico, 2002." Gaceta medica de Mexico 153.Supl. 2 (2017): S5-S12. |
| Not direct health burden result | zu Erbach-Schoenberg, Elisabeth, et al. "Dynamic denominators: the impact of seasonally varying population numbers on disease incidence estimates." Population health metrics 14.1 (2016): 35. |
| Explore association, not burden estimation | Okiring, Jaffer, et al. "Associations between environmental covariates and temporal changes in malaria incidence in high transmission settings of Uganda: a distributed lag nonlinear analysis." BMC Public Health 21.1 (2021): 1962. |
| Risk factor analysis, not burden estimation | Lee, Jung-Seok, et al. "Geographical distribution of risk factors for invasive non-typhoidal Salmonella at the subnational boundary level in sub-Saharan Africa." BMC Infectious Diseases 21.1 (2021): 529. |
| Compare data from two source, not burden estimation | Hamainza, Busiku, et al. "Comparison of a mobile phone-based malaria reporting system with source participant register data for capturing spatial and temporal trends in epidemiological indicators of malaria transmission collected by community health workers in rural Zambia." Malaria journal 13.1 (2014): 489. |
| Hybrid surveillance | Pisharody, Sruti, et al. "Incidence estimates of acute Q fever and spotted fever group rickettsioses, Kilimanjaro, Tanzania, from 2007 to 2008 and from 2012 to 2014." The American journal of tropical medicine and hygiene 106.2 (2021): 494. |
| Case-crossover study and model to examine hazard ratio, not direct burden | Ding, Guoyong, et al. "A mixed method to evaluate burden of malaria due to flooding and waterlogging in Mengcheng County, China: a case study." PloS one 9.5 (2014): e97520. |
| Surveillance reports | Morris, Joan K., et al. "Use of infectious disease surveillance reports to monitor the Zika virus epidemic in Latin America and the Caribbean from 2015 to 2017: strengths and deficiencies." BMJ open 10.12 (2020): e042869. |
| Surveillance reports | Desai, Meghna, et al. "Age-specific malaria mortality rates in the KEMRI/CDC health and demographic surveillance system in western Kenya, 2003–2010." PloS one 9.9 (2014): e106197. |
