# Supplementary material for: Methods for estimating the burden of acute tropical infectious diseases: A scoping review
Source: PLoS Negl Trop Dis. 2026 May 4;20(5):e0013359. doi: 10.1371/journal.pntd.0013359 (PMC13160447; doi:10.1371/journal.pntd.0013359)
Supplement: S6 Table — (DOCX) [file pntd.0013359.s006.docx]

**Table S6 General characteristics of included studies (n = 60).**

|  | Number | Percentage |
| --- | --- | --- |
| Publication year | | |
| Before 2000 | 3 | 5% |
| 2000-2010 | 10 | 17% |
| 2010-2022 | 47 | 78% |
| Study area | | |
| Global | 16 | 27% |
| African Region | 24 | 40% |
| Western Pacific Region | 9 | 15% |
| South-East Asian Region | 12 | 20% |
| Region of the Americas | 9 | 15% |
| Eastern Mediterranean Region | 1 | 2% |
| Disease | | |
| malaria | 21 | 35% |
| dengue | 22 | 37% |
| yellow fever | 8 | 13% |
| chikungunya | 5 | 8% |
| leptospirosis | 3 | 5% |
| scrub typhus | 2 | 3% |
| Japanese encephalitis | 2 | 3% |
| Zika | 1 | 2% |
| typhoid fever | 1 | 2% |
| leishmaniasis | 1 | 2% |
| spotted fever group | 1 | 2% |
| murine typhus | 1 | 2% |
| melioidosis | 1 | 2% |
| schistosomiasis | 1 | 2% |
| Q fever | 1 | 2% |
