# Supplementary material for: Methods for estimating the burden of acute tropical infectious diseases: A scoping review
Source: PLoS Negl Trop Dis. 2026 May 4;20(5):e0013359. doi: 10.1371/journal.pntd.0013359 (PMC13160447; doi:10.1371/journal.pntd.0013359)
Supplement: S1 Fig — (DOCX) [file pntd.0013359.s007.docx]

**S1 Fig. Distribution of modelling approach categories across three time periods (Pre-2010, 2010–2017, 2018–2025) among included studies.**

**
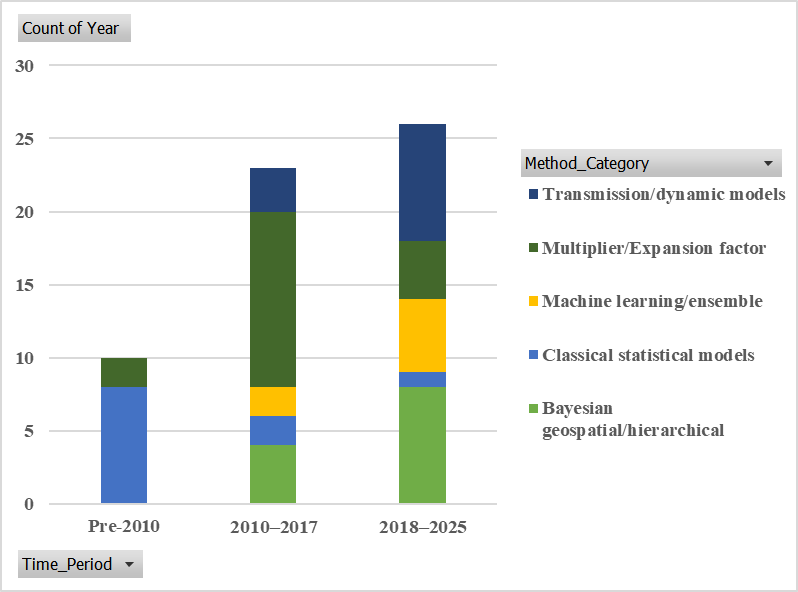
**
