# Supplementary material for: Methods for estimating the burden of acute tropical infectious diseases: A scoping review
Source: PLoS Negl Trop Dis. 2026 May 4;20(5):e0013359. doi: 10.1371/journal.pntd.0013359 (PMC13160447; doi:10.1371/journal.pntd.0013359)
Supplement: Attachment — Submitted filename: Point-by-point response to comments.docx [file pntd.0013359.s009.docx]

**Additional Editor Comments:**

In preparing your revised manuscript for resubmission, please address in full the following comments in addition to the comments from the two reviewers:

1. As raised by both reviewers, and required in item 8 of the PRISMA-ScR Checklist, please ensure you provide in your supplementary material the full electronic search strategy, such that it could be repeated.

We thank the reviewers for highlighting the importance of transparency and reproducibility in accordance with Item 8 of the PRISMA-ScR Checklist. We have now included the complete electronic search strategy in the Supplementary Information as “Table S2: Full search strategy”. This table provides the full PubMed search string, including all keywords, Boolean operators, in sufficient detail to allow replication of the search process.

2. As noted by reviewer 2, the current manuscript title doesn't accurately reflect the scope of diseases considered in your scoping review. Please adjust the title accordingly, e.g. "Methods for estimating the burden of acute *tropical* infectious diseases: a scoping review", and adjust the corresponding wording throughout the manuscript.

We thank the reviewer for this important observation. We agree that the original title could be interpreted as referring to all acute infectious diseases, whereas our inclusion criteria were restricted to acute tropical infectious diseases, primarily those aligned with PLOS Neglected Tropical Diseases and related neglected conditions. The title has been revised to: “Methods for estimating the burden of acute tropical infectious diseases: a scoping review.”

In addition, we have carefully reviewed the manuscript and adjusted the wording throughout the Introduction, Methods, Results, and Discussion sections to consistently reflect the specific scope of acute tropical infectious diseases. We appreciate the reviewer’s suggestion, which has improved the clarity and precision of the manuscript.

3. A key paper is missing from the included studies, which was the first study to apply a formal modelling framework to map the global distribution of dengue disease burden: Bhatt S, Gething PW, Brady OJ et al (2013) Nature. https://doi.org/10.1038/nature12060, which raises a concern about the search strategy and/or screening and assessment process. Please investigate why this paper was not identified and/or included in the scoping review, and whether this indicates any systematic issues with the review methodology.

We thank the reviewer for highlighting the omission of Bhatt et al. (2013), which is indeed a seminal study applying a formal modelling framework to map the global distribution and burden of dengue. Upon re-examination of our screening records, we identified that this study was mistakenly classified during full-text screening as an occurrence/risk mapping study rather than a burden estimation study. As a result, it was excluded under the criterion that studies must explicitly estimate health burden. We acknowledge that this was an error in interpretation during the screening phase.

In response to this comment, we undertook a comprehensive re-review of all full-text screened articles to ensure that no additional studies were misclassified. To enhance transparency and reproducibility, we have now added Table S5 in the Supplementary Information, titled “List of articles excluded at full-text review stage and corresponding justification based on predefined eligibility criteria”. This table provides the full list of excluded full-text articles along with explicit reasons for exclusion based on our predefined criteria.

Following this re-evaluation, the results, figures, and summary statistics have been updated accordingly. We confirm that this issue arose from an isolated misclassification during eligibility assessment and does not reflect a systematic flaw in the search strategy itself. Nevertheless, we have clarified the screening procedures in the Methods section to improve transparency and minimize ambiguity. We appreciate the reviewer’s careful attention, which has strengthened the rigor and transparency of our review.

4. The Global Burden of Disease methodology and the WHO DALYs calculation methodology should be introduced briefly in the introduction, before the subsequent reference in the Methods section to the exclusion of studies that directly applied these methodologies without any additional modelling or methodological advancement (line 118-122)

We thank the reviewer for this helpful suggestion. We agree that briefly introducing the Global Burden of Disease (GBD) framework and the World Health Organization (WHO) DALY calculation methodology in the Introduction improves clarity, particularly given that studies directly applying these standardized approaches without methodological innovation were part of our exclusion criteria. We have now added a concise description of the GBD framework and WHO DALY methodology in the Introduction (Lines 79–95). This addition explains the origin and structure of the DALY metric, including its components, years of life lost (YLL) and years lived with disability (YLD), and clarifies how these standardized frameworks are commonly used in global health burden estimation. The text also provides context for our methodological focus, namely the inclusion of studies that extend or adapt these frameworks rather than those that directly apply them without additional modelling development.

5. Methods Line 103: "The original search was done in 30, March 2024." The word 'original' implies that a second or updated search may have been done later - please provide details of any subsequent search if so, or adjust wording to remove this ambiguity if not. Also, the methods state publications up to 2024 (presumably March) were included, but in results section no publications after 2022 (Fig 2; line 166). If this is correct, please state in results that there were no eligible publications found in 2023-24.

We thank the reviewer for noting the ambiguity regarding the search timeline. In response, we conducted an updated search in October 2025 to ensure that the review reflects the most recent literature. The Methods section has been revised to clarify that the initial search was conducted on 30 March 2024, followed by an updated search in October 2025. The updated search identified 425 additional records published between 2023 and 2025. After title and abstract screening, 25 articles were retained for full-text assessment. Of these, 11 newly identified studies met the inclusion criteria and were incorporated into the final analysis. In addition, prompted by editorial comments regarding potential omissions (including the Bhatt et al. 2013 study), we re-examined all articles previously excluded at the full-text stage and repeated the snowballing process. This comprehensive reassessment led to the inclusion of additional eligible studies that had been previously excluded due to misclassification of burden measures. As a result, the total number of included studies increased from 42 to 60. All screening procedures were repeated for newly identified records, and the Results, figures, and summary statistics have been updated accordingly to reflect the expanded search period. This revision removes ambiguity and ensures that the review includes eligible studies published up to October 2025.

6. Methods Line 125: "(3) the article didn't measure any health burden (e.g., economic cost or social burden)" - this wording is confusing, does it mean to say "e.g. only measured economic cost or social burden"? Please improve the wording.

We thank the reviewer for pointing out the ambiguity in the original wording. We have revised the exclusion criterion. The Methods section now states: “Specific exclusion criteria included: (1) the article did not focus on acute tropical infectious diseases; (2) the article didn’t mention any specific methods; (3) the article only measured economic cost or social burden; or (4) the full text of the article could not be obtained; or the article was not in English.” This revision clarifies that studies were excluded if they exclusively assessed economic or social burden without estimating health burden metrics (e.g., incidence, mortality, DALYs).

7. Figure 1: the last box in the Screening section of the flowchart ('Additional records identified through articles' references') seems like it belongs up in the Identification section, as a secondary means of identifying records that (presumably) then would have been screened and assessed for eligibility in the same way as the 1756 records identified from PubMed. The current flowchart implies that these records were directly included in the review without screening/eligibility assessment.

We thank the reviewer for this important observation regarding the structure of the PRISMA flow diagram. In response, we have revised Figure 1 to ensure that records identified through reference screening are presented within the Identification section of the flowchart, consistent with PRISMA-ScR guidance. In the updated figure, “Additional records identified through articles’ references” is now shown as a secondary source of identification alongside database records, and these records are incorporated into the total number of records screened. All records identified through reference screening underwent the same eligibility assessment as those identified through database searches. The revised flowchart now clearly reflects this process and eliminates any implication that these studies were directly included without screening.

8. Results Line 157: "A total of 1,756 records were identified via the search strategies of PubMed and loaded into Rayyan." Presuming that Rayyan is a software tool, this detail should be provided in the methods, not in the results, and with an explanation of what this software was used for.

Thank you for this helpful comment. We agree that the description of Rayyan, as a screening tool, belongs in the Methods section rather than in the Results. We have therefore removed the reference to Rayyan from the Results section and revised the Methods section accordingly. Specifically, in the subsection Search strategy and study selection (Lines 136–137), we have added the following clarification: “The identified records were imported into Rayyan, a web-based systematic review management tool that enables blinded screening, reviewer conflict resolution, and machine learning–assisted prioritization to streamline study selection.”

9. The use of the term 'prevalence' throughout the manuscript conflates different measures: please be specific in referring to e.g. 'parasite prevalence' for malaria (e.g. line 195), 'dengue seroprevalence' where that is what you mean (e.g. line 208).

Thank you for highlighting this important issue. We have now revised the manuscript to ensure greater precision and clarity. Specifically: For malaria (line 229), we now explicitly refer to “parasite prevalence surveys”, and the sentence reads: “Malaria burden estimation relies on diverse base data, including reported cases from national surveillance systems, parasite prevalence surveys at subnational or community levels, incidence and mortality data derived from household surveys and modelled maps of disease risk.” For dengue (line 245), we now explicitly refer to “dengue seroprevalence data”, and the sentence reads: “Dengue burden estimation similarly relies on a variety of base data, including reported cases from national surveillance systems, dengue seroprevalence data from surveys, incidence and mortality data, as well as age-stratified incidence data derived from cohort studies and literature reviews.”

10. Results Line 196-7: the description of the diverse base data sources used in malaria burden estimation states that this includes "...modelled maps of disease risk (19-36). These data are sourced from a combination of large-scale sources like the Malaria Atlas Project (MAP) (31,36)....". The 'modelled maps of disease risk' produced by MAP and others are not base data, rather they are the modelled burden estimation outputs that are the subject of this scoping review. References #31 and 36 cited here report geospatial modelling estimates of malaria mortality and incidence, respectively. Similarly at line 218 in the discussion of base data sources for dengue burden estimation, "....fine-grained grid-based resolutions (e.g., 5x5 km grids) (49-51)...", these are model-based outputs at 5x5 km pixel resolution, not base data. Improved clarity is needed in the discussion of base data INPUTS (i.e. geolocated data on disease occurrence, parasite prevalence, incidence) versus the modelled maps of disease risk that are the OUTPUTS of disease burden modelling.

Thank you for this important clarification regarding the distinction between empirical base data and model-derived outputs. We agree that our previous wording could have conflated primary empirical inputs (e.g., surveillance case counts, parasite prevalence surveys, seroprevalence data) with model-derived geospatial products (e.g., MAP prevalence surfaces or 5 × 5 km dengue risk grids). In this section, our intention was to describe the types of data used as inputs in the included burden estimation studies. Within these analytical frameworks, both raw empirical observations and previously modelled epidemiological surfaces can function as input layers. However, we recognize that it is methodologically important to distinguish between:

- Empirical base inputs (directly observed epidemiological data), and
- Previously modelled outputs (e.g., geospatial prevalence or risk surfaces), which may serve as secondary input layers in downstream burden estimation frameworks.

We have therefore revised the manuscript to make this hierarchical distinction explicit. We now explicitly state that malaria burden estimation relies on both empirical data inputs and previously modelled outputs, clarifying that MAP products and similar modelled geospatial surfaces are not raw observational data but are sometimes used as covariate inputs or epidemiological layers for subsequent burden calculations (Lines 227-237). Similarly, in the dengue section, we have clarified that high-resolution grid-based estimates represent modelled outputs rather than primary base data, although they may subsequently be used as inputs for downstream burden estimation (Lines 255-258).

11. Table 2. It seems surprising that there were no studies included with estimates of DALYs attributable to malaria. Presumably this is because all such studies met the criteria for exclusion due to direct application of the WHO DALYs calculation method without any modification - whereas more diverse methods were applied for dengue DALY estimation? This warrants mention in the results or discussion section.

Thank you for raising this important point. Following the updated search and inclusion process, our review now includes 11 studies estimating DALYs: six on dengue, one on malaria and four on chikungunya. Table 2 (now Table 1) has been updated accordingly. Although malaria remains the most frequently studied disease overall in terms of incidence and mortality modelling, relatively few included studies estimated malaria DALYs using novel modelling approaches. This likely reflects the dominance of standardized DALY estimation conducted within WHO and GBD frameworks, which were excluded when applied without additional methodological innovation. In contrast, dengue and chikungunya studies more often incorporated expansion factors, seroprevalence-based incidence estimation, or hybrid modelling approaches prior to DALY calculation, leading to greater methodological diversity and inclusion in this review. We have added clarification in both the Results (Lines 275-281) and Discussion sections (Lines 480-487) to explain this distribution.

12. As raised by the reviewers, the Covariates section of the results (including Table 3) is overly descriptive and lacks analytical synthesis. Please make clearer how the consideration of covariates relates to the evolving methodologies for disease burden estimation; i.e. environmental and vector ecology covariates are a critical input for geospatial modeling and machine learning methods, as they inform inference of disease burden in areas without empirical data based on observations in other locations. Whereas other/older approaches based on expansion factors etc may have considered only sociodemographic and/or health system characteristics or even no covariates at all.

Thank you for this valuable suggestion. We agree that the original Covariates section was primarily descriptive. We have revised this section to provide greater analytical synthesis by explicitly linking covariate selection to methodological evolution in burden estimation.

(Lines 378-392) Specifically, we now clarify that earlier approaches relying on expansion factors or multiplier adjustments typically incorporated limited demographic or healthcare-related covariates, whereas modern geospatial and machine learning frameworks integrate environmental and vector ecology variables to enable spatial prediction in areas without empirical data. We emphasize that covariates are central to allowing predictive inference beyond observed locations and represent a methodological shift from adjustment-based estimation to spatially continuous modelling frameworks.

We have also refined Table 2 to clarify how different covariate types function within burden estimation models. Additionally, we reinforce this interpretation in the Discussion section.

13. Items 17 (Results of individual sources of evidence) and 20 (Limitations) in the PRISMA-ScR checklist (Supplementary material) have been marked as N/A, which is not an adequate response. Please address these items in your revised manuscript. Item 17 requires that "For each included source of evidence, present the relevant data that were charted that relate to the review questions and objectives". This should be addressed by including in Supplementary Table S2 the other relevant characteristics that were charted for each study, as described in your methods (lines 130-5), specifically the method for disease burden estimation, base data sources, covariates, and techniques to address uncertainty, for each record. Please also make consistent the format of the 'Authors' column in Table S2, and add the citation number corresponding to the reference list in the main manuscript.

Thank you for highlighting this important issue regarding compliance with the PRISMA-ScR checklist. We have now fully addressed both items in the revised manuscript and supplementary materials.

For Item 17 (Results of individual sources of evidence): We have substantially expanded the Supplementary Information to provide detailed charted data for each included study. Specifically: Table S3 now presents the comprehensive list of included studies with basic characteristics, including Authors, Title, Year, Location conducted, WHO region, Disease studied, Spatial resolution, Spatial extent, and Burden measure. Table S4 now provides detailed methodological information extracted from each study, including Model used, Base data, Base data source, Covariates included, and Uncertainty evaluation techniques. Together, these two tables ensure that all relevant information charted for each included source of evidence is transparently reported and directly aligned with the review objectives.

In addition, we have: Standardized the format of the “Authors” column across supplementary tables. And we have also added the corresponding citation number (as per the main reference list) for each included study to ensure consistency and traceability.

For Item 20 (Limitations): We have added an expanded and comprehensive Limitations section in the Discussion (Lines 511–555), explicitly addressing methodological constraints of the scoping review, including database restriction, language restriction, exclusion of direct GBD/WHO applications, lack of formal quality appraisal, disease imbalance, and the rapidly evolving nature of modelling methodologies.

14. The discussion of the need for improved data sharing and accessibility at lines 432-6 would be strengthened by mentioning existing initiatives to collate publicly available datasets, such as opendengue.org (<https://doi.org/10.1038/s41597-024-03120-7>).

Thank you for this valuable suggestion. We have revised the Discussion section (Lines 580–583) to explicitly include OpenDengue.org as an example of a successful publicly accessible disease data platform. The revised text now reads: “Existing initiatives that collate publicly available disease datasets, such as OpenDengue.org, which provides an openly accessible global dengue surveillance database; the Infectious Diseases Data Observatory (IDDO), which curates and standardizes individual-level infectious disease data; large-scale population survey platforms such as DHS and MICS; and the WHO Global Health Observatory, which compiles country-level health indicators, demonstrate the feasibility and value of such efforts and offer a model for similar platforms for other acute infectious diseases. .”

**Journal Requirements:**

1) Please ensure that the CRediT author contributions listed for every co-author are completed accurately and in full.

At this stage, the following Authors/Authors require contributions: Qian Wang, Richard James Maude, Nicholas Philip John Day, and Benn Sartorius. Please ensure that the full contributions of each author are acknowledged in the "Add/Edit/Remove Authors" section of our submission form.

The list of CRediT author contributions may be found here: <https://journals.plos.org/plosntds/s/authorship#loc-author-contributions>

Thank you for the reminder regarding completion of the CRediT author contribution statements. We confirm that the full CRediT roles for all listed authors have now been completed and accurately entered in the “Add/Edit/Remove Authors” section of the submission system, in accordance with PLOS Neglected Tropical Diseases authorship guidelines.

The contributions are specified as follows:

Qian Wang: Data Curation, Formal Analysis, Funding Acquisition, Investigation, Methodology, Visualization, Writing – Original Draft Preparation

Richard James Maude: Conceptualization, Methodology, Project Administration, Supervision, Writing – Review & Editing

Nicholas Philip John Day: Conceptualization, Project Administration, Supervision, Writing – Review & Editing

Benn Sartorius: Conceptualization, Methodology, Supervision, Writing – Review & Editing

2) Please upload all main figures as separate Figure files in .tif or .eps format. For more information about how to convert and format your figure files please see our guidelines:

We have converted all main figures to .tif format in accordance with the journal’s figure guidelines and uploaded them as separate figure files.

3) Please ensure that the funders and grant numbers match between the Financial Disclosure field and the Funding Information tab in your submission form. Note that the funders must be provided in the same order in both places as well.

State the initials, alongside each funding source, of each author to receive each grant. For example: "This work was supported by the National Institutes of Health (####### to AM; ###### to CJ) and the National Science Foundation (###### to AM).".

If you did not receive any funding for this study, please simply state: u201cThe authors received no specific funding for this work.u201d

Thank you for this clarification. The Funding Source statement now reads: Funding Source: This research was funded in part by the Wellcome Trust [220211] to QW, RJM and ND. BS was supported by an Australian National Health and Medical Research Council Investigator Grant (GNT2034827) and the Operational Research and Decision Support for Infectious Diseases (ODeSI) program, funded by The University of Queensland's Health Research Accelerator (HERA) initiative (2021–2028). The funders had no role in study design, data collection, data analysis, data interpretation, or writing of this study.

Reviewers' Comments:

**Reviewer #1 Dinesh Bhandari**

Introduction

1. A few sentences providing background on the existing burden of two major NTDs, malaria and dengue, in endemic regions, and highlighting how gaps in precise burden estimates have slowed global control and elimination efforts, would strengthen the rationale for this study.

We thank the reviewer for this valuable suggestion. We have revised the Introduction to include a concise paragraph highlighting the current burden of malaria and dengue in endemic regions, with specific reference to their substantial morbidity and mortality and the disproportionate impact on vulnerable populations. We also emphasize how persistent discrepancies between reported and modelled estimates, driven by under-ascertainment, surveillance limitations, and spatial heterogeneity, have complicated intervention targeting, vaccine deployment, and long-term elimination planning. This addition strengthens the rationale for the present scoping review by clearly illustrating why robust and transparent methodological frameworks for burden estimation are critically needed. The revised text has been incorporated into the Introduction (Lines 66–77).

Methods

2. The authors should justify why PubMed was considered an exhaustive source for this review. While PubMed indexes a wide range of biomedical research, it does not capture all studies relevant to the research question. For instance, journals such as Environmental Research Letters publish work on environmental determinants of health outcomes, including infectious diseases, but not all such articles are indexed in PubMed. Standard practice is to include at least one additional database such as Scopus or Web of Science to complement PubMed search outputs. Please explain why other databases were excluded.

We thank the reviewer for this important comment. We agree that searching multiple databases can increase comprehensiveness, particularly in systematic reviews aimed at exhaustive evidence synthesis. In this scoping review, however, our primary objective was to map and characterize methodological frameworks used in estimating the burden of acute tropical infectious diseases rather than to conduct a quantitative synthesis of all relevant empirical findings. We therefore focused on PubMed, which provides broad and well-established coverage of biomedical, epidemiological, and global health journals where infectious disease burden modelling studies are predominantly published. To ensure transparency and reproducibility, we have now provided the full PubMed electronic search strategy and list of excluded studies with reasons in the Supplementary Materials. We have also clarified our rationale for database selection in the Methods section (Lines 125-128) and explicitly acknowledged, in the Limitations section (Lines 541-547), that restricting the search to PubMed and English-language publications may have resulted in the omission of some studies indexed in other interdisciplinary or environmental science databases. We believe this clarification strengthens the transparency of our methodological approach while remaining consistent with scoping review guidance.

3. Why was the initial screening conducted by only one researcher? This approach may introduce bias. Both JBI and Cochrane recommend at least two researchers for initial screening, with an additional reviewer involved for conflict resolution when necessary.

We appreciate this comment and agree that dual screening can enhance reproducibility. In this scoping review, our objective was to map and characterize methodological approaches rather than to conduct a quantitative synthesis or critical appraisal of effect estimates. Title/abstract and full-text screening were conducted by one reviewer (QW) using predefined inclusion and exclusion criteria to ensure consistency in interpretation. A second reviewer (RJM) independently reviewed uncertain or ambiguous records, and any disagreements were resolved through discussion. To improve transparency, we have revised the Methods section (Lines 131-134) to clearly describe this screening process. There are methodological research suggest that targeted single-reviewer screening, particularly when predefined exclusion criteria are clearly operationalized and ambiguous records are verified by a second reviewer, results in minimal loss of sensitivity and can be efficient and acceptably accurate under defined conditions(1).

Nevertheless, we acknowledge in the Limitations section (Lines 563-566) that single-reviewer screening may introduce some risk of missed eligible studies, although predefined criteria and secondary verification were applied to mitigate this risk. We believe this clarification strengthens the methodological transparency of the review while remaining consistent with accepted scoping review practices.

4. The authors state that the study design was selected to address specific research questions. However, explicit reference to a standard scoping review framework, such as the Population, Concept, and Context (PCC) framework, would strengthen the robustness of the study design.

We thank the reviewer for this helpful suggestion. We agree that explicitly anchoring the study design within a recognized scoping review framework strengthens methodological transparency. We have now clarified in the Methods section (lines 107-114) that this review was guided by the Population–Concept–Context (PCC) framework. Specifically, the Population comprised populations affected by acute tropical infectious diseases; the Concept focused on methodological approaches used for disease burden estimation; and the Context encompassed all geographic regions and settings. We have added this clarification to the Study Design section to strengthen alignment with established scoping review methodology.

5. Please include the full search strategy in the supplementary material. This would enhance transparency and support future researchers in replicating or extending the study.

We thank the reviewer for this important suggestion. To enhance transparency and reproducibility, we have now included the complete electronic search strategy in the Supplementary Material (Table S2). The full PubMed search string is provided exactly as implemented, including all search terms and Boolean operators. We have also clarified this in the Methods section (line 124-124) to ensure that future researchers can replicate or extend the search strategy.

6. The manuscript notes that a biomedical information specialist (librarian) contributed to protocol development. However, I did not find a reference to the protocol itself. If I have overlooked it, please clarify; otherwise, it is recommended that the review protocol be made publicly available and cited to strengthen the rigor of the work.

We thank the reviewer for this valuable suggestion. To enhance transparency and methodological rigor, we have now registered the review protocol on the Open Science Framework (OSF) (Registration DOI: 10.17605/OSF.IO/FSZ5B ). We have added the protocol registration and DOI to the Methods section of the manuscript (Lines 118-119).

Results

7. If possible, reconsider the color palette in Figure 2. Specifically, the colors used to represent dengue and malaria, as well as JE and Q fever, are difficult to distinguish at a glance. If possible, a network diagram (for example, a Sankey diagram) would be a useful way to summarize the results. The diagram could show how study regions link to the NTDs examined, then to the models or methods used, and finally branch out to the parameters included in the models or outcomes reported (such as mortality, incidence, or DALYs).

We thank the reviewer for this helpful suggestion. We have updated Figure 2 to improve visual discrimination by (a) using a colourblind-friendly, higher-contrast palette and (b) combining low-frequency diseases (overall occurrence < 2 across the dataset) into a single category labelled “Others”. This reduces the number of colours-classes and substantially improves legibility while preserving the meaningful disease-specific patterns. We considered producing a Sankey/network diagram; however, given the heterogeneity of methods and the modest number of studies per method–disease cell, a single Sankey would have been overly complex and reduced interpretability. If the editors would prefer, we can prepare a supplementary Sankey that shows Region- Disease- Broad method groups (e.g., Bayesian geostatistics, machine learning, multiplier approaches) for ease of visualization.

8. While the main paper includes a section to summarise the models used for burden estimation, supplementary materials should also include a column specifying the model or method used for estimation in each selected study. At present, this information is missing.

We thank the reviewer for this helpful suggestion. We have substantially expanded the Supplementary Information to provide detailed charted data for each included study. Specifically, Table S3 now presents the comprehensive list of included studies with their basic characteristics, including Authors, Title, Year, Location conducted, WHO region, Disease studied, Spatial resolution, Spatial extent, and Burden measure. In addition, we have created a new Table S4, which provides detailed methodological information extracted from each study, including the specific model or analytical framework used for burden estimation, base data and data sources, covariates included, and uncertainty evaluation techniques.

Discussion

9. In the results section, the authors note issues with extrapolation in data-scarce regions, often based on simplistic assumptions that ignore local context. This is particularly relevant to the GBD strategy from IHME, which has been criticized for missing contextual nuances and sometimes producing inaccurate results. This issue could be elaborated further, especially in the paragraph preceding the recommendations section. Highlighting the fallacy of overly simplistic assumptions and the overuse of extrapolation, rather than marking use of available sources to generate primary estimates would enrich the discussion.

We thank the reviewer for this insightful comment. We have expanded the Discussion section (lines 494-508) to more explicitly address the methodological challenges associated with extrapolation in data-scarce settings. The revised text highlights how reliance on simplified assumptions and cross-regional extrapolation may overlook local epidemiological, healthcare, and socio-economic heterogeneities, potentially introducing systematic bias in burden estimates.

10. While this review successfully maps existing methods for estimating the burden of acute infections, the discussion could be more analytical rather than purely descriptive. The recommendations, in particular, could be framed more equitably by emphasizing partnerships with local policymakers and scientific experts. The authors identify data-related challenges and suggest improvements focused largely on data collection, management, and sharing. However, local institutional capacity development should also be considered. While it may be unrealistic to expect every institution in these settings to have expert modelers or epidemiologists, systems could be established where experts from resource-rich contexts support the development of platforms based on advanced modeling strategies. Local institutions could then input relevant parameters and receive burden estimates in a clear, interpretable form. Such an approach could mitigate ethical concerns around external data use/sharing and foster equitable partnerships between local institutions and supporting collaborators.

We thank the reviewer for this thoughtful and constructive suggestion. We have revised the Recommendations section (lines 641-652) to incorporate a more analytical discussion of equitable partnerships and local institutional capacity development. Specifically, we now emphasize the importance of collaborative modelling frameworks, co-development of analytical tools, and context-sensitive capacity building to ensure that burden estimation efforts are locally relevant and ethically grounded. This addition highlights the need for sustainable partnerships between local institutions and international research groups, moving beyond purely technical recommendations toward more equitable and context-aware approaches.

**Reviewer #2**

This scoping review delivers a timely and comprehensive map of how acute NTD burden has been estimated over the past three decades. Its strengths lie in the systematic collation of methodological trends, the clear geographic and disease distributions, and the transparent reporting of covariates and uncertainty techniques. Below are some topic-by-topic suggestions for improvement.

Introduction

The title suggest that the scoping review covers acute infectious diseases broadly. However, the inclusion criteria in the Methods section specify that only acute infectious diseases listed in the NTDs recognized by PLOS Neglected Tropical Disease are considered. This limitation must be clearly stated in the Introduction or Methods to avoid misleading readers into thinking the review covers all acute infectious diseases (e.g., COVID-19, influenza, Ebola).

We thank the reviewer for this important clarification. To avoid any ambiguity regarding scope, we have revised the title to “Methods for estimating the burden of acute tropical infectious diseases: a scoping review.” We have also clarified in both the Introduction and Methods sections that this review focuses specifically on acute tropical infectious diseases within the NTD framework and does not encompass all acute infectious diseases (e.g., COVID-19, influenza, Ebola).

Method

1.Search Strategy Insufficiently Comprehensive: The search was limited to PubMed, potentially omitting relevant studies indexed in other major databases such as Web of Science, Embase, or Scopus. Additionally, there is no mention of whether grey literature (e.g., government reports) was searched, which is particularly important for capturing unpublished or locally conducted research.

We thank the reviewer for this important methodological comment. We acknowledge that searching multiple databases and grey literature sources can enhance comprehensiveness, particularly in systematic reviews aimed at exhaustive evidence synthesis. In this scoping review, however, our objective was to map and characterize methodological approaches used in peer-reviewed burden estimation studies rather than to provide a complete census of all burden estimates or policy reports.

We therefore focused on PubMed, which provides broad coverage of biomedical, epidemiological, and global health journals where infectious disease burden modelling studies are predominantly published. We did not include grey literature sources (e.g., government or institutional reports), as these often lack detailed methodological transparency required for extraction of modelling frameworks and analytical approaches.

To ensure transparency, we have clarified the rationale for database selection in the Methods section (Lines 125-128) and explicitly acknowledged the restriction to PubMed and peer-reviewed English-language publications as a limitation in the Discussion (Lines 542-547). We recognize that relevant studies indexed in other interdisciplinary databases or unpublished reports may not have been captured.

2.The manuscript states that search strings covered "outcomes," "methods," and "diseases," but the actual search terms or a full search query (e.g., for PubMed) are not provided. Including the full search strategy as supplementary material is essential for transparency and reproducibility.

We thank the reviewer for this important suggestion. To enhance transparency and reproducibility, we have now included the complete electronic search strategy in the Supplementary Material (Table S2). The full PubMed search string is provided exactly as implemented, including all search terms and Boolean operators. We have also clarified this in the Methods section (line 124) to ensure that future researchers can replicate or extend the search strategy.

3.Incomplete Reporting of Screening Process: While the manuscript includes a PRISMA flowchart and numbers at each stage, it does not describe quality control measures during data extraction, such as whether dual independent screening and extraction were conducted.

We thank the reviewer for this important comment and agree that transparent reporting of screening and extraction procedures strengthens methodological rigor. As this study was conducted as a scoping review, the primary aim was to map methodological approaches rather than perform a quantitative synthesis.

Title/abstract and full-text screening were conducted by one reviewer (QW) using predefined inclusion and exclusion criteria to ensure consistency in interpretation. A second reviewer (RJM) independently reviewed uncertain or ambiguous records, and any disagreements were resolved through discussion. To improve transparency, we have revised the Methods section (Lines 131-134) to clearly describe this screening process. There are methodological research suggest that targeted single-reviewer screening, particularly when predefined exclusion criteria are clearly operationalized and ambiguous records are verified by a second reviewer, results in minimal loss of sensitivity and can be efficient and acceptably accurate under defined conditions(1).

Nevertheless, we acknowledge in the Limitations section (Lines 563-566) that single-reviewer screening may introduce some risk of missed eligible studies, although predefined criteria and secondary verification were applied to mitigate this risk. We believe this clarification strengthens the methodological transparency of the review while remaining consistent with accepted scoping review practices.

4.Although scoping reviews do not require formal quality assessment, a brief descriptive summary or commentary on the methodological quality of included studies would help readers interpret the strength of the evidence.

We thank the reviewer for this valuable suggestion. Based on the detailed methodological information extracted and presented in Supplementary Table S4, we have now added a descriptive methodological robustness summary in the Discussion section (lines 517-530). This analysis highlights variation in uncertainty quantification, model validation practices, and transparency of assumptions across modelling approaches. This addition provides readers with contextual insight into the relative methodological strength of included studies while remaining consistent with scoping review guidance.

Results

1.The manuscript included a study from 1987 (B.M. Greenwood et.al.) in Table S2, but Table 1 (Publication year distribution) only covers studies from 1998 onwards. Furthermore, the Methods section states that the search was conducted in PubMed for publications from 1990 to 2024. Please clarify how the 1987 study was identified and included.

We thank the reviewer for this careful observation. The PubMed database search was restricted to publications from 1990 onwards. However, the 1987 study by Greenwood et al. was identified through reference screening (“snowballing”) of an included article (Gething et al., 2016, Mapping plasmodium falciparum mortality in Africa between 1990 and 2015.) and was subsequently assessed for eligibility. As illustrated in the PRISMA flow diagram (figure 1), studies identified through reference screening underwent the same screening and eligibility assessment process as database-identified records. We have clarified this in the Methods section (lines 128-130) to explicitly distinguish between the database search time restriction and additional records identified through reference screening. Furthermore, the publication year distribution table has been revised (now Table S6) to categorize studies published before 2000, ensuring consistency with the inclusion of the 1987 study.

2.Figure 2 and Table 1 appear to contain overlapping information regarding the distribution of included studies by publication year. We recommend integrating this information into a single, clear visual or moving one of them to the supplementary materials to avoid repetition and enhance the flow of the results section.

We thank the reviewer for this helpful suggestion. To reduce redundancy and improve the flow of the Results section, we have moved the detailed summary table of publication year distribution to the Supplementary Materials (now Table S6). Figure 2 is retained in the main text as the primary visual summary of temporal and regional distribution. The publication year distribution is now described concisely in the text, while detailed counts are provided in the supplementary table. This revision eliminates overlap and improves clarity.

3.The section on covariates is a useful list but lacks analysis. A brief discussion on how the selection of covariates is rationalized for different diseases and settings, and the relative importance of different covariate types (environmental, demographic, healthcare-related).

We thank the reviewer for this constructive suggestion. We have now added an analytical paragraph at the end of the Covariates section in the Results (lines 407-421) to explain how covariate selection varies according to disease ecology and modelling framework. Specifically, we highlight the predominance of environmental and vector-related covariates in geospatial models of vector-borne diseases, and the greater reliance on demographic and healthcare-related variables in multiplier-based approaches aimed at correcting underreporting. We have also briefly reinforced this interpretation in the Discussion to emphasize the methodological implications of covariate integration.

4.The manuscript claims that burden-estimation methods have shifted from traditional surveillance to advanced approaches such as machine-learning and Bayesian geospatial models, yet this evolution is described only qualitatively. No quantitative evidence—e.g., counts or proportions of studies using each method type by time period, a stacked bar chart, or trend test—is provided.

We thank the reviewer for this valuable suggestion. To provide quantitative support for the observed methodological evolution, we grouped methods into five broad methodological classes: multiplier/expansion factor approaches, classical statistical models, Bayesian geospatial or hierarchical models, machine learning/ensemble methods, and transmission or dynamic models based on the approaches identified across included studies. This classification was developed inductively to facilitate structured comparison of modelling paradigms rather than to impose a pre-existing formal taxonomy. Their distribution across three time periods was checked and the results are presented in a new Supplementary Figure (Figure S1), which demonstrates a clear shift from multiplier-based and classical regression approaches in earlier years toward Bayesian geospatial, ensemble, and dynamic modelling frameworks in more recent studies. This addition quantitatively substantiates the narrative description of methodological progression in the manuscript.

Discussion

1.Disease coverage was highly skewed: malaria and dengue accounted for 76 % of all included studies, whereas other NTDs (e.g., leptospirosis, Melioidosis) were represented by only a handful of cases. This imbalance markedly limits the generalisability of the findings, yet the manuscript does not discuss how it may have shaped the overall conclusions.

We thank the reviewer for this important observation. We have revised the Limitations section to explicitly acknowledge that the dominance of malaria and dengue studies shapes the methodological patterns identified in this review (lines 555-563). In particular, the prominence of geospatial and Bayesian modelling frameworks largely reflects developments within these high-burden vector-borne diseases. We now clarify that methodological insights for less-studied acute tropical infectious diseases may be underrepresented, and that this imbalance limits the generalisability of our conclusions across all diseases within scope.

2.Table 4 repeats well-known imperatives such as "Data sharing and accessibility" and "Apply machine learning". Indicate which of these actions are new or prioritised for acute NTDs. I recommend strengthening specifying actionable public health measures.

We thank the reviewer for this constructive feedback. We have substantially revised this table (now Table 3) to provide more targeted and actionable recommendations specific to acute tropical infectious diseases. The revised table now distinguishes priority levels (immediate, medium-term, structural), specifies disease-relevant methodological gaps (e.g., overreliance on extrapolation, limited uncertainty propagation), and emphasizes practical public health actions such as strengthening sentinel surveillance, requiring probabilistic uncertainty reporting, and supporting shared modelling platforms with local institutional capacity building. These revisions move beyond general methodological statements and provide more operational guidance aligned with the findings of this review.

3.As a scoping review, the inclusion criteria—limited to English-language, peer-reviewed articles—may introduce language and publication biases. Relevant non-English regional studies or grey literature containing unique estimation methods might have been excluded. Furthermore, restricting the search to PubMed alone, without including other major databases such as Embase or Scopus, could lead to the omission of significant literature. It is recommended to address these potential "literature selection biases" in the Limitations section and discuss their possible impact on the representativeness of the conclusions.

We thank the reviewer for highlighting this important issue. We have revised the Limitations section (lines 541-547) to explicitly acknowledge potential language, publication, and database selection biases arising from restricting inclusion to English-language, peer-reviewed studies indexed in PubMed. We now discuss how these constraints may affect the representativeness of the mapped methodological landscape, particularly for underrepresented regions and studies published outside mainstream biomedical journals.

Conclusion

While the Conclusion provides a general summary of the study’s contributions and future directions, it would benefit from a more explicit integration of key results presented in the manuscript.

We thank the reviewer for this suggestion. We have now added a dedicated Conclusion section at the very end of the discussion (lines 666-680) that explicitly synthesizes the key findings of the review, including the dominance of malaria and dengue studies, the methodological evolution from multiplier-based to Bayesian and geospatial modelling approaches, shifts in covariate integration, and variation in uncertainty quantification practices. The revised Conclusion now also clearly articulates the key priorities emerging from our findings, including strengthening locally grounded surveillance systems, fostering equitable modelling partnerships and capacity development in endemic settings, and promoting greater standardization and transparency in uncertainty reporting and modelling assumptions. This addition more clearly integrates the main results and reinforces the overall contributions of the review.

Reference:

1.	Nama N, Hennawy M, Barrowman N, O’Hearn K, Sampson M, McNally JD. Successful incorporation of single reviewer assessments during systematic review screening: development and validation of sensitivity and work-saved of an algorithm that considers exclusion criteria and count. Systematic Reviews. 2021;10(1):98.
