# Supplementary material for: Methods for estimating the burden of acute tropical infectious diseases: A scoping review
Source: PLoS Negl Trop Dis. 2026 May 4;20(5):e0013359. doi: 10.1371/journal.pntd.0013359 (PMC13160447; doi:10.1371/journal.pntd.0013359)
Supplement: Attachment — Submitted filename: Point-by-point response to comments - round2.docx [file pntd.0013359.s010.docx]

Additional Editor Comments :

Thank you for addressing the two reviewers' comments and additional editor's comments very thoroughly in your revised manuscript. There are only a few minor additional points requiring your attention, in order for the manuscript to be accepted for publication:

1. Table S5: this is a valuable addition. However there is a switch halfway through the table from providing the full citation details and explicit reasons for exclusion, to instead providing only the paper title and a nondescript reason for exclusion. Please revise to include the full information for all excluded publications listed in Table S5

Thank you for this helpful comment. We have updated all entries to include the full citation details (authors, year, and journal) along with clear and explicit reasons for exclusion for each publication in Table S5.

2. Line 130: "The original search was done in 30, March 2024" -> "The original search was done on 30 March 2024"

Thank you for noting this. We have corrected the wording to “The original search was done on 30 March 2024” at Line 130 of the revised manuscript.

3. Table 3: update table title to align with revised manuscript title 'Table 3. Key Recommendations for Advancing Acute *Tropical* Infectious Disease Burden Estimation'

Thanks for point this out. We have updated the title of Table 3 to align with the revised manuscript title as recommended at Line 640.

4. Regarding Reviewer 1's comment #10, one minor addition is suggested at line 646:

"Collaborative platforms that allow local institutions to input region-specific parameters, adapt assumptions to local epidemiology, and retain ownership of *data inputs and* analytical outputs are therefore critical."

Thanks for your suggestion. We have incorporated the suggested wording “data inputs and” at line 649.

5. It is recommended that you cite the new supporting information Figure S1 at an appropriate place in the manuscript text, though it is not a journal requirement that supporting information is cited.

Thank you for this suggestion. We have now cited Supporting Information Figure S1 in the revised manuscript (Results, “Method development over time” section, line 336). The figure is referenced in the opening sentence to support the description of temporal trends in modelling approaches.
